# Overexpression of *TCERG1* as a prognostic marker in hepatocellular carcinoma: A TCGA data-based analysis

**DOI:** 10.3389/fgene.2022.959832

**Published:** 2022-10-10

**Authors:** Pan Yang, Huaifeng Liu, Yan Li, Qunwei Gao, Xin Chen, Junyan Chang, Yangyang Li, Shuran Chen, Rui Dong, Huazhang Wu, Changqing Liu, Gaofeng Liu

**Affiliations:** ^1^ School of Life Science, Anhui Province Key Laboratory of Translational Cancer Research, Bengbu Medical College, Bengbu, China; ^2^ Department of Gynecologic Oncology, First Affiliated Hospital of Bengbu Medical College, Bengbu, Anhui, China; ^3^ Department of Gastrointestinal Surgery, First Affiliated Hospital of Bengbu Medical College, Bengbu, Anhui, China

**Keywords:** biomarker, gene set enrichment analysis, hepatocellular carcinoma, TCERG1, prognosis

## Abstract

**Objective:** Transcription elongation factor 1 (*TCERG1*) is a nuclear protein consisted of multiple protein structural domains that plays an important role in regulating the transcription, extension, and splicing regulation of RNA polymerase II. However, the prognostic and immunological role of *TCERG*1 in human cancer remains unknown. In this study, we analyzed the expression of *TCERG1* gene in hepatocellular carcinoma (HCC) patients, its clinical significance, and its possible prognostic value by bioinformatics.

**Methods:** RNA sequencing data and clinicopathological characteristics of patients with HCC were collected from TCGA and CCLE databases. The Wilcoxon rank-sum test was used to analyze the expression of *TCERG1* in HCC tissues and normal tissues. The protein levels of *TCERG1* between normal and liver cancer tissues were analyzed by the Human Protein Atlas Database (HPA) (www.proteinatlas.org). Validation was performed using the Gene Expression Omnibus (GEO) dataset of 167 samples. The expression of *TCERG1* in HCC cells were verified by qRT-PCR, and CCK-8, scratch assay and Transwell assay were performed to detect cell proliferation, migration and invasion ability. According to the median value of *TCERG1* expression, patients were divided into high and low subgroups. Logistic regression, GSEA enrichment, TME, and single-sample set gene enrichment analysis (ssGSEA) were performed to explore the effects of *TCERG1* on liver cancer biological function and immune infiltrates. *TCERG1* co-expression networks were studied through the CCLE database and the LinkedOmics database to analyze genes that interact with *TCERG1*.

**Results:** The expression levels of *TCERG1* in HCC patient tissues were significantly higher than in normal tissues. Survival analysis showed that high levels of *TCERG1* expression were significantly associated with low survival rates in HCC patients. Multifactorial analysis showed that high *TCERG1* expression was an independent risk factor affecting tumor prognosis. This result was also verified in the GEO database. Cellular experiments demonstrated that cell proliferation, migration and invasion were inhibited after silencing of *TCERG1* gene expression. Co-expression analysis revealed that *CPSF6* and *MAML1* expression were positively correlated with *TCERG1*. GSEA showed that in samples with high *TCERG1* expression, relevant signaling pathways associated with cell cycle, apoptosis, pathways in cancer and enriched in known tumors included Wnt signaling pathway, Vegf signaling pathway, Notch signaling pathway, MAPK signaling pathway and MTOR pathways. The expression of *TCERG1* was positively correlated with tumor immune infiltrating cells (T helper two cells, T helper cells).

**Conclusion:** *TCERG1* gene is highly expressed in hepatocellular carcinoma tissues, which is associated with the poor prognosis of liver cancer, and may be one of the markers for the diagnosis and screening of liver cancer and the prediction of prognosis effect. At the same time, *TCERG1* may also become a new target for tumor immunotherapy.

## Introduction

Hepatocellular carcinoma is a common primary liver cancer that more occurs in male patients and has a high morbidity and mortality rate. According to the World Health Organization in 2020, the incidence and mortality rates of HCC ranked seventh and fourth, respectively, and both gradually increased with age. According to statistics, about 50% of patients are in China, which has become a serious threat to patient health and safety (Chen et al., 2016; Harding et al., 2018; Hasan et al., 2020). The onset of HCC is a multifactorial, multi-step complex process influenced by dual factors of the environment and the patient itself, but the molecular mechanism by which it occurs and develops remains unclear (Hu et al., 2018). Among them, hepatitis B virus (HBV), hepatitis C virus (HCV) infection, aflatoxin, obesity, metabolic syndrome, and genetic factors are the more prominent risk factors (Jindal et al., 2019).

The common clinical treatment strategies for HCC are surgical resection, chemotherapy, radiotherapy, radiofrequency ablation, *etc.* Ultrasound (US) and serum alpha-fetoprotein (AFP) are common diagnostic methods for it, but the diagnostic results still lack specificity and sensitivity (Wang and Wei, 2020). Patients with HCC have no obvious early symptoms, and most of them are diagnosed at advanced stages or have distant metastases and have lost the opportunity for radical surgery (Hasan et al., 2020). In order to better diagnose early HCC, new biomarkers have become a hot spot for research. The biological and clinical diversity of HCC poses a great challenge for individualized clinical treatment (Jeng et al., 2015; Forner et al., 2018; Thorsson et al., 2018).

Although advances have been made in the treatment and diagnosis of hepatocellular carcinoma in the last decade, its survival prognosis is poor and 5-year survival rates remain unsatisfactory (Kulik and El-Serag, 2019; Chen et al., 2020). The pathogenesis of HCC is extremely complex, involving cell cycle regulation, signal transduction and other processes. This reflects the different functions and multiple interactions of many genes (Craig et al., 2020). Therefore, exploring the specific mechanisms of hepatocarcinogenesis and analyzing the prognostic factors of hepatocellular carcinoma may be helpful in improving the prognosis of patients and finding effective therapeutic targets (Quaresma et al., 2015).

Transcriptional extension regulator 1 (*TCERG1*) is a highly conserved human nuclear protein, formerly known as CA150, that contains multiple protein domains, specifically three WW domains at the amino terminal and six consecutive FF domains at the carboxyl terminal. The transcriptional elongation factor used to regulate *HIV-1 Tat* gene activation was originally discovered from the nuclear extract fraction of HeLa cells. Subsequently, it was identified in highly purified spliceosomes (Sune et al., 1997; Neubauer et al., 1998; Makarov et al., 2002; Lin et al., 2004; Deckert et al., 2006). TCERG1 is localized at the splicing factor-rich nuclear speckle interface and nearby transcriptional sites (Sanchez-Alvarez et al., 2006), and is involved in a variety of events including pre-mRNA splicing regulation and regulation of RNA polymerase II transcriptional elongation. Transcriptomic studies showed that *TCERG1* is highly expressed in the brain (hippocampus, cortex, lateral ventricles and cerebellum). Interestingly, *TCERG*1 is involved in the pathogenesis of Huntington’s disease (HD) (Arango et al., 2006; Andresen et al., 2007) and plays a neuroprotective role in HD due to its overexpression that can rescue neuronal cell death caused by mutant HTT neurotoxicity. *TCERG1* has also been identified as a genetic regulator of Tau neurotoxicity (Blard et al., 2007). Many studies have found (Wang et al., 2000; Smith et al., 2004) that *TCERG1* is involved in the regulation of apoptosis. It sensitizes cells to apoptotic agents and thus promotes apoptosis by regulating the variable splicing of apoptotic genes *Bcl-x* and *Fas/CD95*. There are indications that the effect of *TCERG1* on apoptosis also involves changes in mitochondrial membrane permeability (Montes et al., 2015).

At present, the role of *TCERG1* gene in cancer has been reported less, but the clinical pathological significance and systematic analysis of *TCERG1* gene in liver cancer patients have not been reported. Therefore, this study was conducted to assess the prognostic significance of *TCERG1* gene expression in HCC by bioinformatics analysis of clinical features and survival data from The Genome Atlas of Liver Cancer (TCGA-LIHC).

## Material methods

**FIGURE 1:**
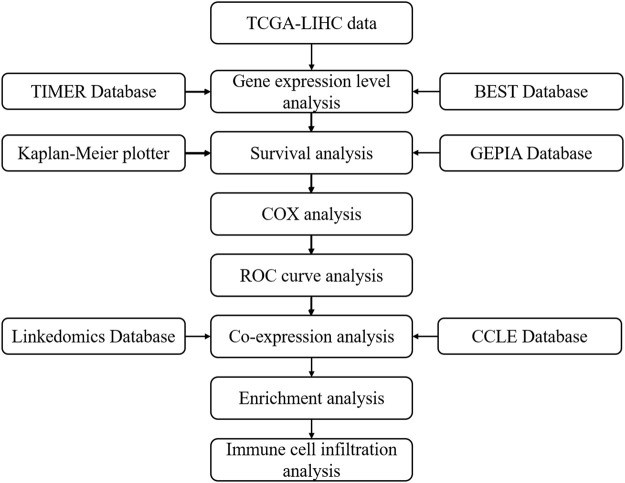
The flowchart of the study.

The flowchart of the study is shown in Figure 1.

### Data collection

The expression data of RNA-seq of hepatocellular carcinoma (TCGA-LIHC) (HTSeq-FPKM was selected from Workflow Type column) and clinical information were downloaded from TCGA database (https://portal.gdc.cancer.gov/). A total of 50 normal tissue samples and 374 tumor tissue samples were obtained. RNA-seq transcriptome sequencing data (transcriptome corrected expression) were also downloaded from the CCLE database for analysis. In addition, the gene expression and clinical information file GSE76427 was downloaded from the Gene Expression Omnibus database (GEO) (https://www.ncbi.nlm.nih.gov/geo/) (Laurent et al., 2011).

### *TCERG1* gene expression analysis

Firstly, we analyzed *TCERG1* expression levels in different types of human cancers by TIMER database (https://cistrome.shinyapps.io/timer/) and GEPIA database (http://gepia2.cancer-pku.cn/#analysis). Secondly, the expression levels of *TGERG1* in hepatocellular carcinoma also verified using the online web tool BEST (https://rookieutopia.com/app_direct/BEST/). The BEST website contains over 50,000 samples from 27 solid tumors and is divided into eight main modules that explore gene expression, prognosis, functional characteristics, immunotherapy, immune infiltration, immunomodulators, genomic mutations, and even the development of small molecule drugs, while the TCGA-LIHC data downloaded from TCGA (Normal group N = 50, tumor group T = 374) were normalized by using the limma and beeswarm packages in R software (version 4.1.2), and the *TCERG1* expression in the tumor and normal groups were extracted for mapping. The patient samples were further screened and analyzed, and the samples from the same patients in the paracancer group were paired with those in the tumor group (50 pairs in total).

### Survival analysis

*TCERG1* gene expression and overall survival (OS) of 370 TCGA-LIHC patients were analyzed using the SURVIVAL package in R software. The Kaplan Meier (http://kmplot.com/analysis) (Menyhart et al., 2018) and the GEPIA online database (Tang et al., 2017) were used to validate the survival curve analysis of the *TCERG1* gene.

### Clinical diagnostic value analysis

We removed the patient data with incomplete clinical information by combining the patient’s age, sex, grade, TNM staging and distant metastasis from the relevant clinical data of liver cancer patients downloaded, and finally obtained the data of 329 patients. The patients were divided into high and low groups according to the median value of *TCERG1* gene expression. We used the survival ROC package for ROC curve plotting analysis and obtained the area under the curve (AUC). Potential prognostic factors were screened using univariate Cox analysis, multivariate Cox regression to analyze the effects of *TCERG1* expression and other clinical variables on total survival (OS).

### Co-expression analysis

To further investigate the molecular mechanisms associated with *TCERG1*, the LinkedOmics database (http://linkedomics.org/login.php) (Zhou et al., 2019) was used to identify *TCERG1* co-expression genes by pearson and the results were displayed by heat map. Using the Limma package in R software, 489 co-expressed genes were obtained by filtering with a person coefficient >0.4 and *p* < 0.01. Finally, the co-expressed genes were sorted by cor value, and 10 genes were taken for each of low and high expression (only mRNA was analyzed) and heat map was plotted using the ggplot2 package in R software. Finally, the top 50 genes associated with *TCERG1* in both databases were selected to take the intersection for further analysis.

### Enrichment analysis

TCGA-LIHC data (expression with duplicate genes removed using the Limma package in R software) were subjected to GSEA enrichment analysis based on the median value of *TCERG1* gene expression. 1,000 gene set alignments were performed for each analysis. Enrichment pathways with NES >1, NOM *p*-value < 0.05 and FDR *q*-value < 0.25 were considered to be statistically significant. GO and KEGG functional analyses were also performed for *TCERG1* and its co-expressed genes *CPSF6* and *MAML1*.

### Analysis of *TCERG1* in immune cells

The TISIDB database (http://cis.hku.hk/TISIDB/index.php), a website that integrates multiple types of data resources into tumor immunology (Ru et al., 2019), collects a large number of human cancer datasets from the TCGA database. The correlation between *TCERG1* expression in HCC by immune or molecular subtypes was explored through the TISIDB database. *p* values less than 0.05 were considered to be statistically significant. Single sample gene set enrichment analysis (ssGSEA) was performed on tumor samples from 24 immune cell types using the GSVA software package (Bindea et al., 2013; Hanzelmann et al., 2013).

### Cell culture

LO2, SMMC-7721, SNU-449, HUH-7 and MHCC97-H cells were cultured in DMEM medium (Gibco, ThermoFisher Scientific, United States) with 10% fetal bovine serum (Life Technologies, United States), 1% penicillin and streptomycin (100u/ml penicillin and 100 mg/ml streptomycin of Life Technologies) in an incubator humidified air at 37°C with 5% CO_2_.

### Quantitative real-time (qRT) PCR assay

Total RNA was extracted from cells according to instructions using the TRIzol (Invitrogen) method. Contaminated genomic DNA was removed with RQ1 DNase (Promega, Madison, WI, american). Reverse transcription of total RNA into cDNA according to the TAKARA kit reaction system. qRT-PCR was quantitatively analyzed using SYBR. Primer: hTCERG1-fw:ggcgagagtgaacgattcaac. hTCERG1-rv:tggaggaggtcgcatcagag. hGAPDH-fw:actttggtatcgtggaaggactcat. hGAPDH-rv:gtttttctagacggcaggtcagg.

### Western blot

Normal control (NC) group and interfering group (*Si-TCERG1-1, si-TCERG1-2*) cells were collected and lysed on ice for 30 min with appropriate amounts of RIPA and PMSF, and protein quantification by BCA. Separated by SDS-PAGE electrophoresis, transferred to PVDF membrane, closed for 2 h, followed by primary antibody (1:1000) overnight at 4°C and incubated with secondary antibody for 2 h the next day for development analysis and detection of protein bands.

### Cell proliferation assay

CCK8 experiments were divided into NC group and interference group (*Si-TCERG1-1, si-TCERG1-2*), SMMC-7721 and Huh-7 cells after gene regulation were inoculated in 96-well plates at 2000/well, with three replicate wells/group, and 10ul/well CCK-8 reagent was added at 0 h, 24 h, 48 h and 72 h, respectively, and the cells were incubated at 37°C for 2 h, and the absorbance value A450 nm was detected to observe the cell proliferation.

### Wound healing scratch test

Hepatocellular carcinoma cells were inoculated in a six-well plate and scratched with a 10ul short shotgun tip after 24 h. The scratched cells were removed by washing with PBS and incubated with serum-free medium. The cell gaps were photographed with microscope, and the migration was observed after 0 h and 24 h. The cells were analyzed by ImageJ software.

### Cell migration and invasion assay

The cells of each group were digested with trypsin and evenly seeded in Transwell chambers, 800ul of complete culture medium was added to the lower chamber, and after 24 h of incubation, the cells of Transwell chambers were fixed with methanol for 30 min, stained with crystal violet for 20 min, washed with PBS for floating color, and the number of migrated cells was counted after air-drying. For cell invasion assay, Matrigel was added to the upper chamber of Transwell, and after it solidified, cells were seeded in the upper chamber coated with Matrigel, and 800 ul of complete medium was added to the lower chamber and incubated at 37°C for 24 h. The number of cell invasion was detected in the same way.

### Statistical analysis

Statistical analyses and plots were performed using R software (version 4.1.2) and GraphPad software. *t* test, Mann-Whitney *U* test were used were performed to compare between the two groups. Wilcoxon s rank sum test and logistic regression were used to analyze the correlation between clinical characteristics of HCC and *TCERG1* expression. *p* values less than 0.05 were regarded as statistically significant. Univariate and multivariate Cox proportional risk models were used to assess survival analysis. The Spearman rank correlation coefficient method was used to analyze the relationship between *TCERG1* and the level of immune cell infiltration. In addition, statistical significance of cell line experiments was assessed by *t*-test in GraphPad Prism version8 software. The difference was considered to be statistically significant at ∗*p* < 0.05, ∗∗*p* < 0.01, ∗∗∗*p* < 0.001.

## Results

### Identification of *TCERG1* differential expression in human cancers

**FIGURE 2:**
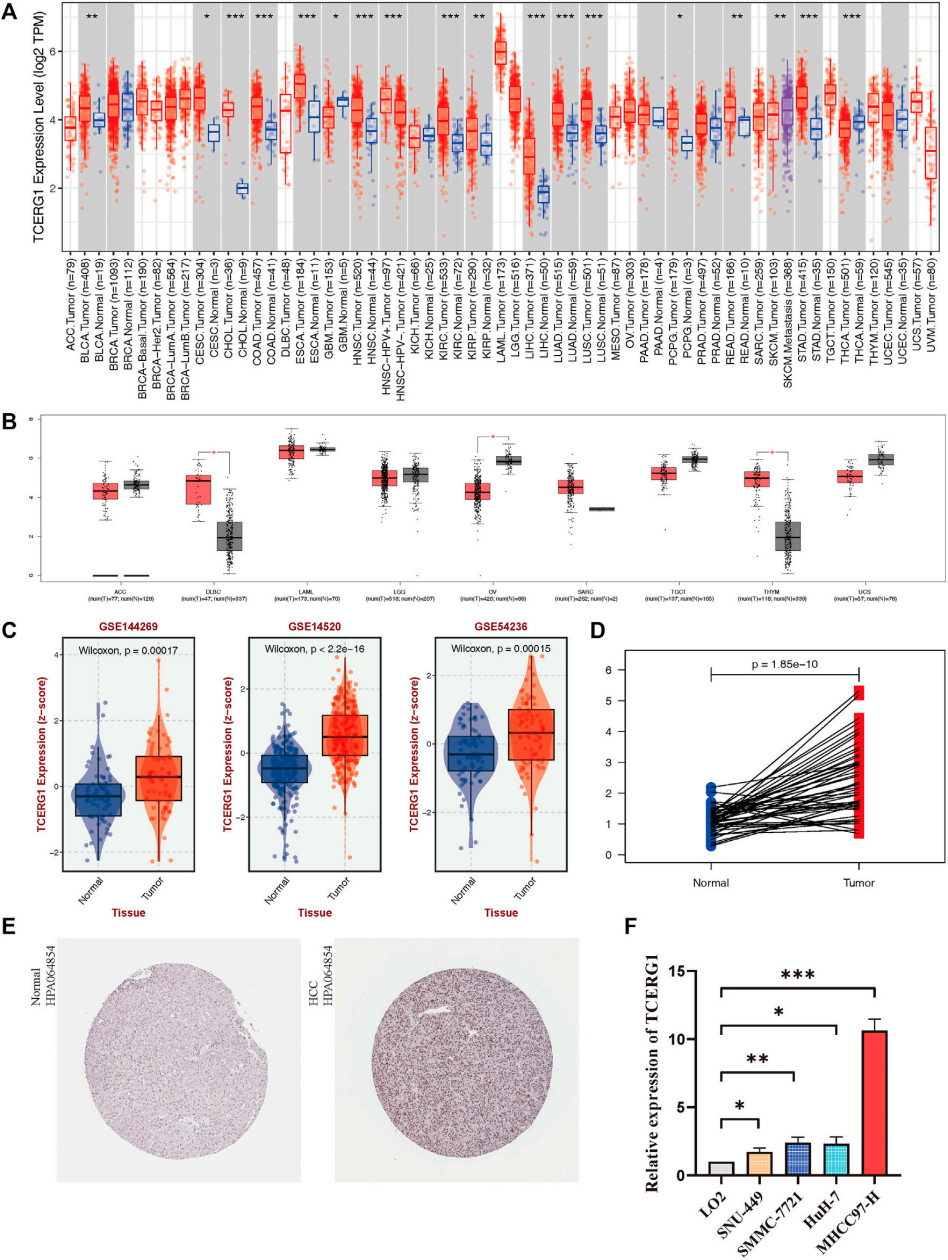
Expression level of *TCERG1* in tumors.**(A)** TIMER database analysis of *TCERG1* expression levels in different cancer types in the TCGA database; **(B)** *TCERG1* expression in several cancers and paired normal tissues in the GEPIA database; *TCERG1* **(C)** 50 pairwise analysis of *TCERG1* gene expression in the paracancer group *versus* the tumor group; *TCERG1* **(D)** *TCERG1* gene expression in liver cancer tissues and normal liver tissues in BEST database **(E)** Immunohistochemical analysis of TCERG1 protein expression in HPA database normal and HCC tissues. Normal liver cancer tissue - patient id: 3402, antibody: HPA064854, staining: not detected, intensity: weak, quantity: <25%; tumor tissue - patient id: 5031, antibody: HPA064854 Staining: medium, intensity: moderate, quantity: >75%; **(F)** mRNA expression levels of *TCERG1* in HCC cell lines. Three independent experiments were performed. (*p < 0.05, **p < 0.01, ***p < 0.001).

To explore the role of *TCERG1* in the clinical prognosis of HCC patients, we first analyzed the expression of *TCERG1* in different cancer types. The results of TIMER database showed that *TCERG1* gene was significantly high expression in BLCA, CHOL, COAD, ESCA, HNSC, KIRC, KIRP, LIHC, LUAD, LUSC, READ STAD and other cancers, while it was low expression in GBM and THCA **(** Figure 2A **)**. The results of GEPIA database analysis showed that they are supplementary results of several cancers without normal tissue pairing in TIMER database **(** Figure 2B **)**. The results suggested that the dysregulation of *TCERG1* expression may be involved in the occurrence and development of various tumors.

### *TCERG1* gene is highly expressed in HCC

We analyzed the expression level of *TCERG1* gene in HCC by the BEST and TCGA databases. The results showed that the expression levels of *TCERG1* gene was significantly higher in LIHC than in normal tissues (*p* = 2.497e-21, *p* = 1.85e-10) (Figure 2C-D, Supplementary Table S2). In addition, immunohistochemical analysis obtained by HPA showed that *TCERG1* had higher expression levels in tumor tissues compared to non-tumor tissues (Figure 2G), while qRT-PCR further verified that *TCERG1* gene was highly expressed in HCC (Figure 2H). These results implied that *TCERG1* overexpression might play an important role in the occurrence and development of HCC.

### Clinical correlation analysis of *TCERG1* expression in HCC patients

**FIGURE 3:**
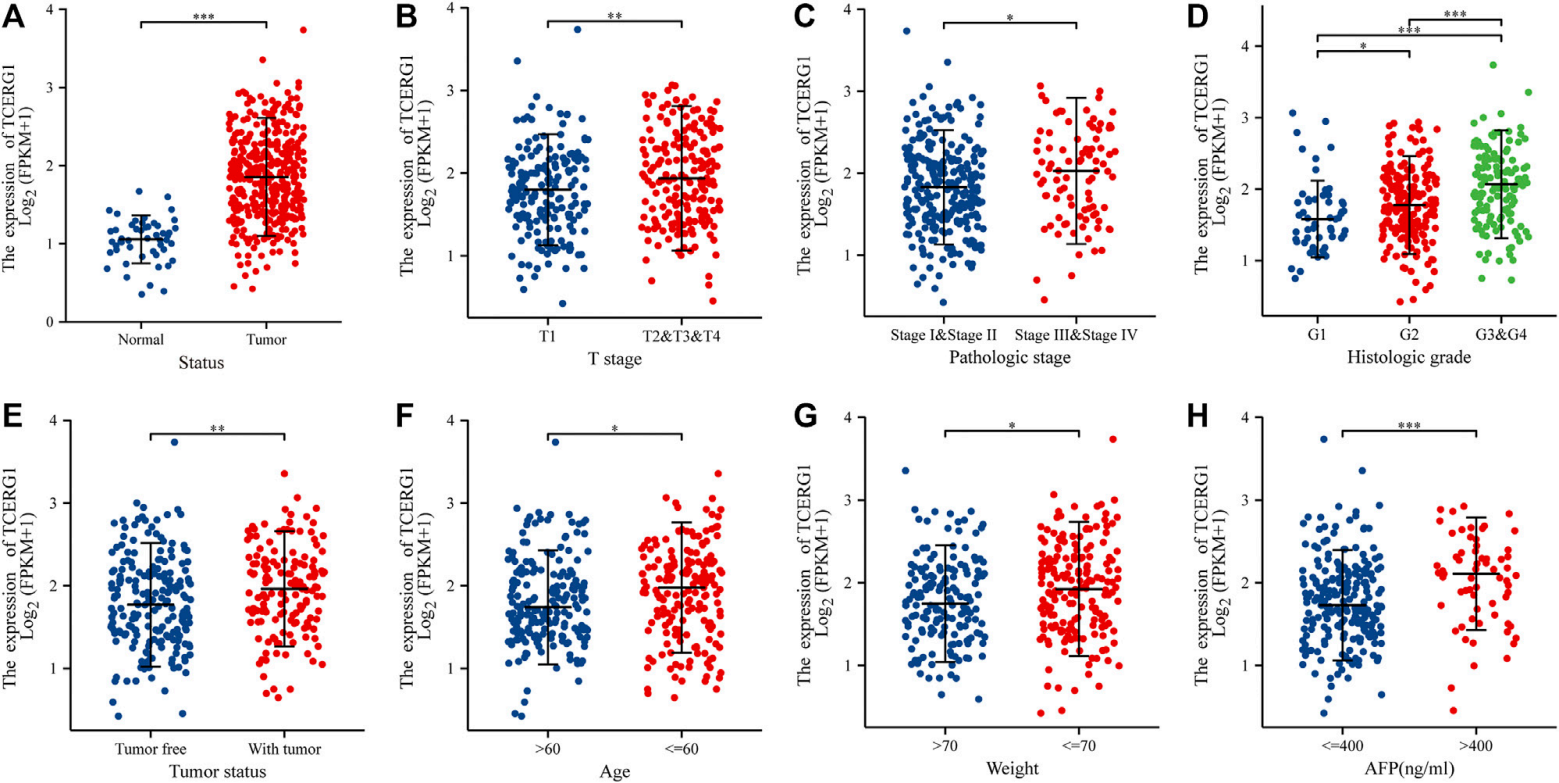
Correlation between *TCERG1* gene expression and clinicopathological characteristics. **(A)** Clinical status; **(B)** T stage; **(C)** Pathologic stage; **(D)** histologic grad; **(E)** tumor status; **(F)**age; **(G)** weight; **(H)** AFP levels.

In order to explore the correlation between *TCERG1* gene and clinical characteristics, we analyzed the prognostic role of *TCERG1* gene in HCC combined with the relevant clinical data in TCGA database. The results showed that *TCERG1* overexpression was significantly correlated with clinical status, T stage, pathologic stage, histologic grade, tumor status, age, weight, and AFP (ng/ml) (Figure 3, Supplementary Table S3) (*p < 0.05, **p < 0.01, ***p < 0.001).

Univariate analysis of logistic regression showed that *TCERG1* gene expression was a categorical dependent variable associated with clinical characteristics of poor prognosis (Table 1). High *TCERG1* expression level was associated with T stage (*p* = 0.048), gender (*p* = 0.039), race (*p* = 0.042), age (*p* = 0.001), and weight (*p* = 0.018), Pathologic stage (*p* = 0.042) Tumor status (*p* = 0.007) and histologic grad correlated (*p* < 0.001). These results indicate that high expression of *TCERG1* is an independent risk factor affecting overall survival in patients with HCC.

**TABLE 1:** Univariate logistic regression analysis of *TCERG1* and clinicopathological characteristics.

Characteristics	Total (N)	Odds ratio (OR)	*p* value
T stage (T2&T3&T4 vs. T1)	368	1.513 (1.004–2.286)	**0.048**
N stage (N1 vs. N0)	256	2.600 (0.328–52.950)	0.411
M stage (M1 vs. M0)	270	0.309 (0.015–2.449)	0.312
Gender (Male vs. Female)	371	0.631 (0.406–0.976)	**0.039**
Race (Black or African American&White vs. Asian)	359	0.647 (0.424–0.983)	**0.042**
Age (>60 vs. ≤60)	370	0.508 (0.335–0.766)	**0.001**
Weight (>70 vs. ≤70)	344	0.597 (0.388–0.913)	**0.018**
Height (≥170 vs. < 170)	339	0.664 (0.429–1.024)	0.065
BMI (>25 vs. ≤25)	335	0.776 (0.504–1.192)	0.247
Pathologic stage (Stage III&Stage IV vs. Stage I&Stage II)	347	1.660 (1.023–2.718)	**0.042**
Residual tumor (R1&R2 vs. R0)	342	1.611 (0.619–4.474)	0.337
Tumor status (With tumor vs. Tumor free)	352	1.794 (1.173–2.757)	**0.007**
AFP(ng/ml) (>400 vs. ≤400)	278	2.863 (1.610–5.228)	**<0.001**
Vascular invasion (Yes vs. No)	315	1.355 (0.851–2.163)	0.201
Histologic grade (G3&G4 vs. G1&G2)	366	2.904 (1.871–4.554)	**<0.001**

**FIGURE 4:**
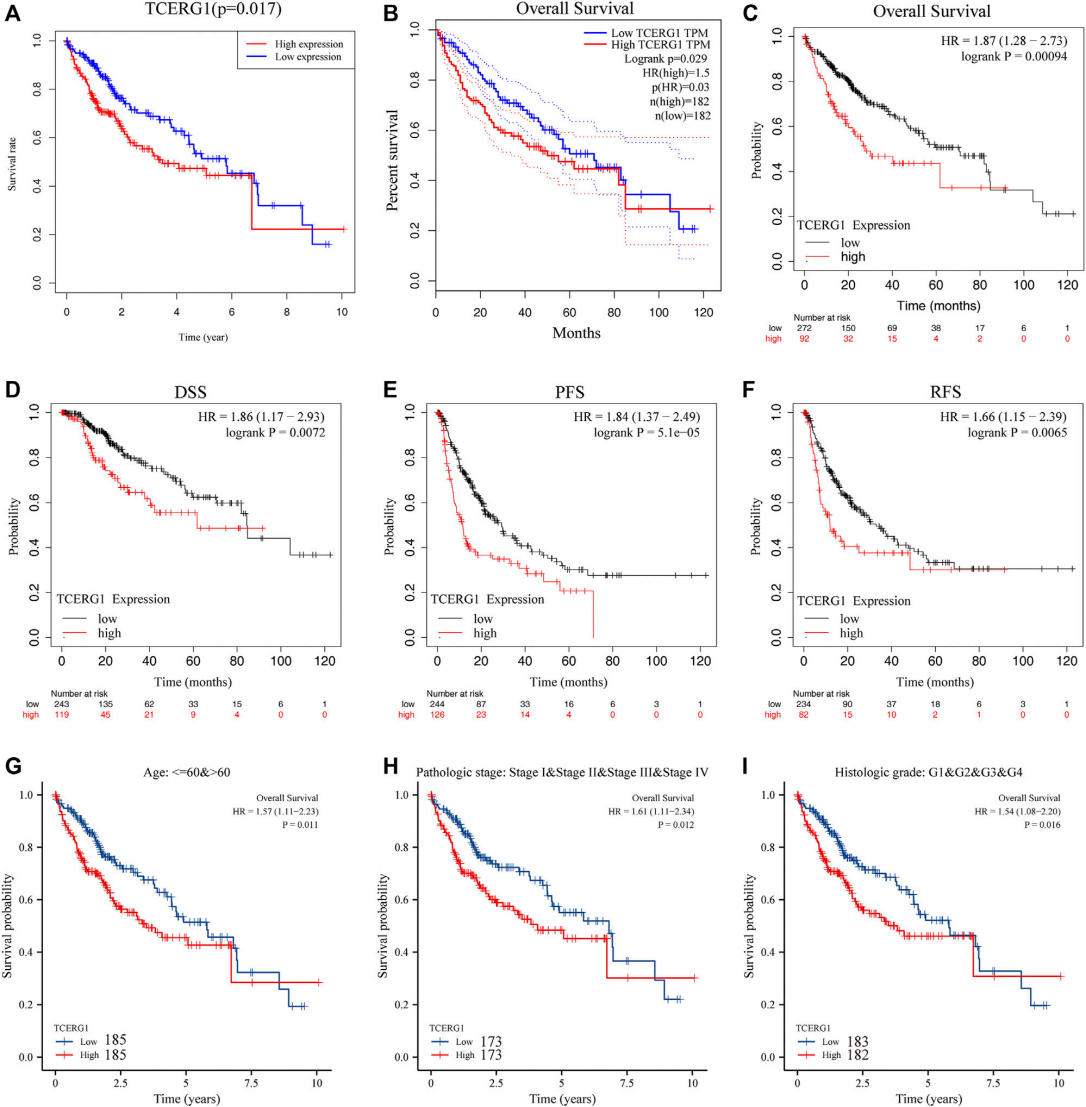
Survival analysis of *TCERG1* gene expression in HCC patients from TCGA-LIHC database. **(A)**TCGA database of *TCERG1* expression level in HCC in relation to OS; **(B)**GEPIA database analysis of *TCERG1* expression level in HCC patients in relation to OS; **(C–F)** Kaplan-Meier website analysis of *TCERG1* expression level in HCC patients in relation to OS, DSS, PFS, RFS; **(G–I)**.age, Pathologic stage, histologic grade OS: overall survival; DSS: disease-specific survival; PFS: progression-free survival; RFS: disease-free survival.

To determine the relationship between *TCERG1* expression levels and prognosis, we plotted the survival curves of *TCERG1* gene by combining clinical data from HCC patients (N = 370) in TCGA. The analysis showed that high *TCERG1* expression and poor prognosis were associated (*p* = 0.017) (Figure 4A). Subsequently, the survival analysis of *TCERG1* gene was further validated using GEPIA and Kaplan Meier databases to obtain OS (*p* = 0.029) and OS (*p* = 0.00094), respectively (Figures 4B,C); It was also found that DSS (*p* = 0.0072), PFS (*p* = 5.1e-05) and RFS (*p* = 0.0065) were significantly lower in the *TCERG1* high expression group than in the low expression group, The results indicate that patients with low expression had better survival, while patients with high expression had significantly lower survival (Figures 4.D–F). Subgroup analysis of different clinical characteristics showed that high *TCERG1* expression and poor prognosis were significantly correlated in age (*p* = 0.011), Pathologic stage (*p* = 0.012), histologic grade (*p* = 0.011, Figures 4G–I). In HCC, high *TCERG1* expression and shortened survival time and poor prognosis were positively correlated, indicating that *TCERG1* could be used as an indicator of disease progression and prognosis in HCC patients.

### Identification of factors influencing the prognosis of HCC

**FIGURE 5:**
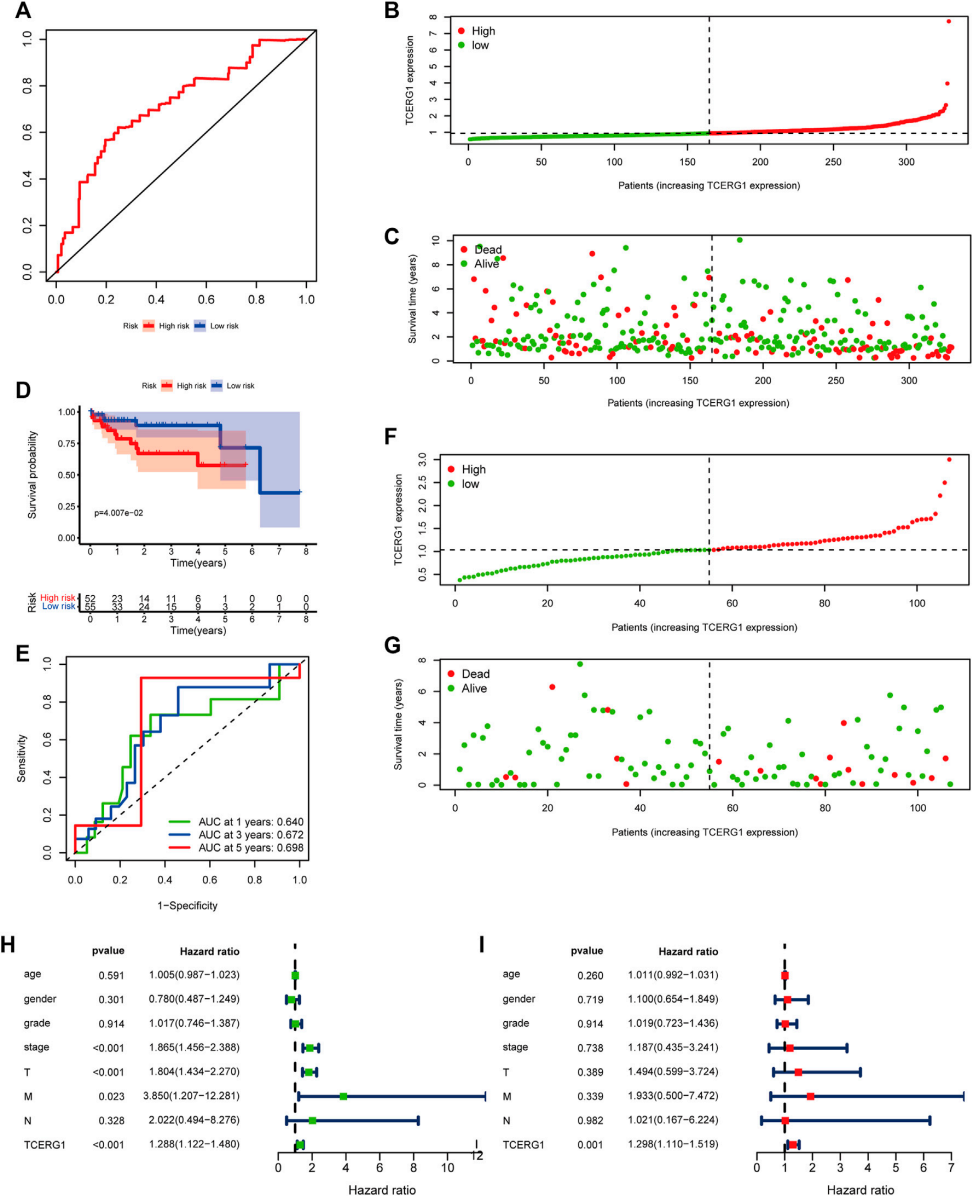
Prognostic analysis of *TCERG1* in HCC.**(A)** ROC curve analysis; **(B,C)** Risk score distribution and survival status of *TCERG1* in HCC; **(D–G)** Prognostic analysis of TCERG1 in HCC in the GEO dataset; **(H)** Univariate Cox regression analysis of risk scores regarding OS in HCC; **(I)**Multivariate cox analysis.

We performed ROC curve analysis on *TCERG1* gene expression data to assess the diagnostic value of this gene. The AUC value is within the range [0.5–1.0](AUC = 0.716), which has some clinical diagnostic value (Figure 5A). The risk score and survival status are shown in Figure 5B,C. The negative prognostic impact of higher TCERG1 expression was validated in the GEO database (GSE76427 Figure ). Meanwhile, we obtained data information from 240 patients for Univariate COX analysis, and the results showed that the expression of *TCERG1* gene in patients with hepatocellular carcinoma (HR = 1.29; 95% CI =1.12–1.48; *p* < 0.001), stage (HR = 1.86; 95% CI = 1.46–2.39; *p* < 0.001), T (HR = 1.80; 95% CI = 1.43–2.27; *p* < 0.001) and M (HR = 3.84; 95% CI = 1.21–12.28; *p* = 0.023) were also high risk factors. Similarly, the results of multivariate Cox regression analysis showed that the expression level of *TCERG1* gene (HR = 1.30; 95% CI = 1.11–1.52; *p* = 0.001) was still independently associated with OS (Figures 5H,I) In conclusion, *TCERG1* gene could be an independent prognostic factor for patients with HCC.

### Co-expression analysis of *TCERG1* gene

**FIGURE 6:**
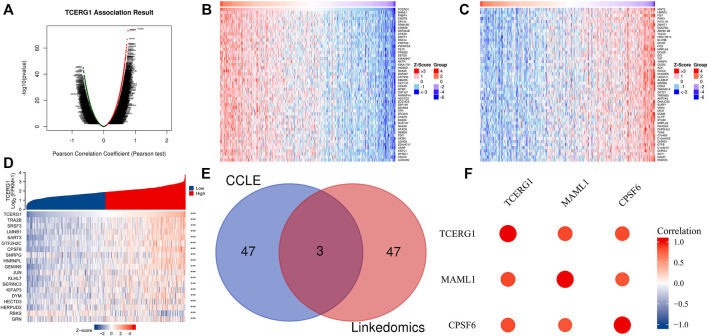
Co-expressed genes of *TCERG1* in HCC. **(A)** LinkedOmics database analysis of co-expressed genes of *TCERG1* in HCC; **(B,C)** heat map of the top 50 positive co-expressed genes (B) and negative co-expressed genes with *TCERG1* (C); **(D)** CCLE database analysis of the top 10 positively and negatively associated co-expressed genes; **(E)** Venn diagram of significantly associated genes from LinkedOmics and CCLE databases; **(F)** *CPSF6*, *MAML1* and *TCERG1* are positively correlated.

To further investigate the possible role of *TCERG1* in hepatocarcinogenesis, we next performed data mining through Linkedomics and CCLE databases to screen for proteins interacting with *TCERG1*. The results of Linkedomics database showed that 7,726 genes were significantly positively correlated with *TCERG1* in HCC and 3245 genes were negatively correlated (FDR <0.01). The heat map shows the top 50 genes positively and negatively associated with *TCERG1* (Figures 6A–C). The CCLE database results showed the top 10 genes positively and negatively associated with *TCERG1* genes in the heat map (mRNA only analysis) (Figure 6D, Supplementary Table S6, *p* < 0.01). Finally, the intersection of the top 50 genes significantly associated with *TCERG1* in both databases revealed that *CPSF6* and *MAML1* were present in both databases, and the results showed that *CPSF6* and *MAML1* were positively associated with *TCERG*1 (Figures 6E,F). In addition, it has been shown that high expression of *CPSF6* and *MAML1* in HCC is associated with poor patient prognosis and they might serve as a potential prognostic marker to predict hepatocarcinogenesis (Wang et al., 2016; Tan et al., 2021). These results above suggested that *TCERG1* and its co-expressed genes *CPSF6* and *MAML1* might be jointly involved in hepatocarcinogenesis, leading to a lower survival rate in patients with hepatocellular carcinoma.

### GO and KEGG analysis

**FIGURE 7:**
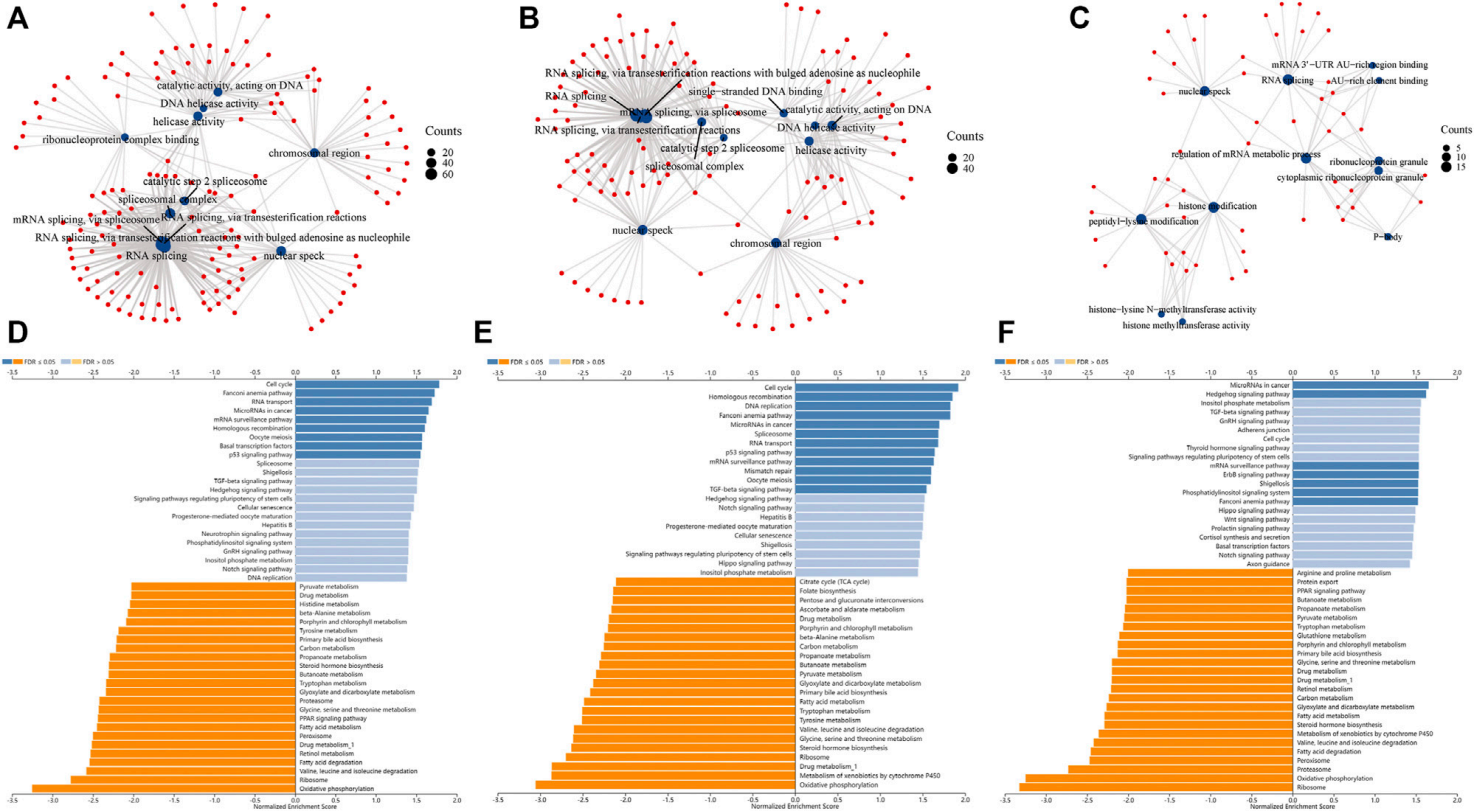
GO and KEGG functional analysis of *TCERG1* and its co-expression genes *CPSF6* and *MAML1*. **(A–C)** GO analysis of *TCERG1*, *CPSF6* and *MAML1* in HCC; **(D–F)** KEGG analysis of *TCERG1*, *CPSF6* and *MAML1* in HCC.

Next, gene set enrichment analysis (GSEA) was used to determine the major gene ontology (GO) of *TCERG1* co-expressed genes. *TCERG1*, *CPSF6*, *MAML1* and their co-expressed genes were found to be enriched in RNA splicing, nuclear speck, chromosomal region, and ribonucleoprotein complex binding (Figures 7A–C). Then, we performed Kyoto Encyclopedia of Genes and Genomes (KEGG) pathway analysis in Linkedomivs database, and the results showed that *TCERG1*, *CSPF6*, *MAML1* were all enriched in Cell cycle, Fanconi anemia pathway, MicroRNAs in cancer, Spliceosome and TGF-β signaling pathway, and ErbB signaling pathway (Figures 7D,E). These results suggested that *TCERG1* and its co-expressed genes *CPSF6* and *MAML1* might be jointly involved in the development of hepatocarcinoma.

### TCERG1 correlation signal path analysis is based on GSEA

**FIGURE 8:**
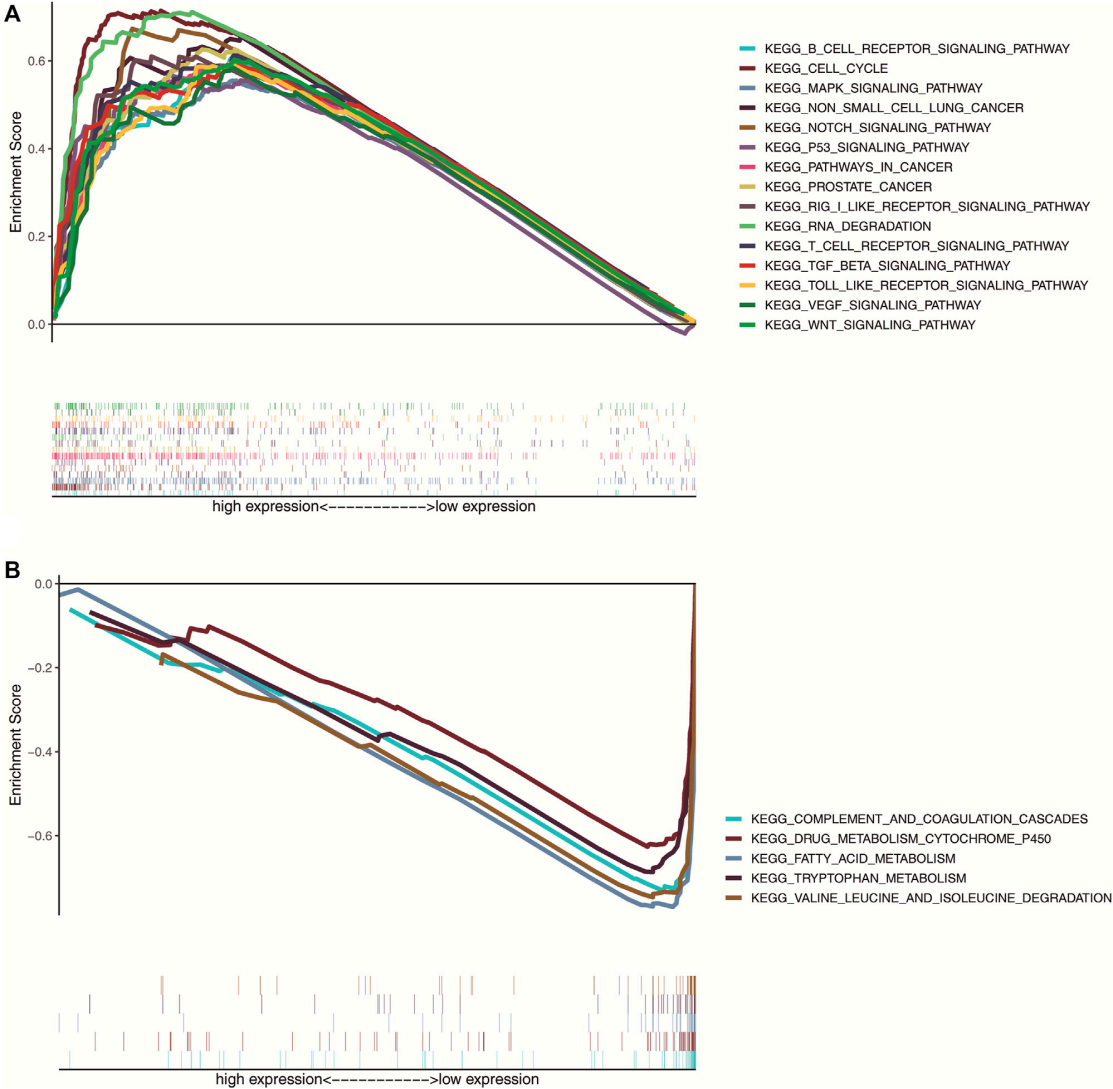
Pathway enrichment analysis of *TCERG1* gene. **(A)** 15 important signaling pathways associated with high *TCERG1* expression; **(B)** five important signaling pathways associated with low *TCERG1* expression (NOM *p* < 0.05 and FDR *q* < 0.05).

To investigate the potential function of *TCERG1* gene in HCC, we divided TCGA-LIHC clinical patient data samples into high and low expression groups according to the median value of *TCERG1* gene expression. Then, we performed GSEA enrichment score analysis to identify the signaling pathways that are differentially activated between high and low *TCERG1* gene expression phenotypes in HCC. Analysis of the enrichment results showed that 134 of 178 signaling pathways were upregulated and 44 were downregulated, of which 88 were upregulated and nine were downregulated (NOM *p* < 0.05). The results showed that the samples with high *TCERG1* gene expression were significantly enriched in cancer-related signaling pathways such as cell cycle, apoptosis and pathways in cancer, as well as signaling pathways known to be associated with hepatocellular carcinoma such as Wnt signaling pathway, Vegf signaling pathway, Notch signaling pathway, MAPK signaling pathway and MTOR signaling pathway (Figure 8). The high *TCERG1* phenotype was also enriched in immune cell-related signaling pathways such as B cell, T cell, *etc.* Complement and coagulation cascades, fatty acid metabolism, tryptophan metabolism, valine leucine and isoleucine degradation, drug metabolism cytochrome P450, and retinol metabolism were enriched in the low *TCERG1* phenotype (Figure 8B; Table 2). These suggested that *TCERG1* might be involved in immunomodulation and play potential role in the occurrence and development of liver cancer.

**TABLE 2:** Gene set of *TCERG1* expression phenotype.

Name	ES	NES	NOM *p*-val	FDR *q*-val
KEGG_PROSTATE_CANCER	0.63	2.03	0	0.003
KEGG_RNA_DEGRADATION	0.71	2.02	0	0.003
KEGG_CELL_CYCLE	0.71	2.02	0	0.003
KEGG_NON_SMALL_CELL_LUNG_CANCER	0.66	2.01	0	0.003
KEGG_PATHWAYS_IN_CANCER	0.59	2.01	0	0.002
KEGG_WNT_SIGNALING_PATHWAY	0.6	2	0	0.003
KEGG_VEGF_SIGNALING_PATHWAY	0.59	1.97	0	0.003
KEGG_NOTCH_SIGNALING_PATHWAY	0.67	1.97	0	0.003
KEGG_MAPK_SIGNALING_PATHWAY	0.56	1.95	0	0.003
KEGG_APOPTOSIS	0.62	1.93	0	0.003
KEGG_TOLL_LIKE_RECEPTOR_SIGNALING_PATHWAY	0.59	1.88	0.002	0.006
KEGG_P53_SIGNALING_PATHWAY	0.57	1.86	0.002	0.007
KEGG_B_CELL_RECEPTOR_SIGNALING_PATHWAY	0.61	1.8	0.004	0.011
KEGG_T_CELL_RECEPTOR_SIGNALING_PATHWAY	0.61	1.79	0.004	0.012
KEGG_COMPLEMENT_AND_COAGULATION_CASCADES	-0.74	-2.04	0	0.002
KEGG_FATTY_ACID_METABOLISM	-0.78	-1.95	0.002	0.012
KEGG_TRYPTOPHAN_METABOLISM	-0.7	-1.94	0.006	0.009
KEGG_VALINE_LEUCINE_AND_ISOLEUCINE_DEGRADATION	-0.75	-1.91	0.01	0.012
KEGG_DRUG_METABOLISM_CYTOCHROME_P450	-0.63	-1.88	0.006	0.016
KEGG_RETINOL_METABOLISM	-0.63	-1.83	0.008	0.022

### Correlation of *TCERG1* gene expression levels and immune infiltrating cells

**FIGURE 9:**
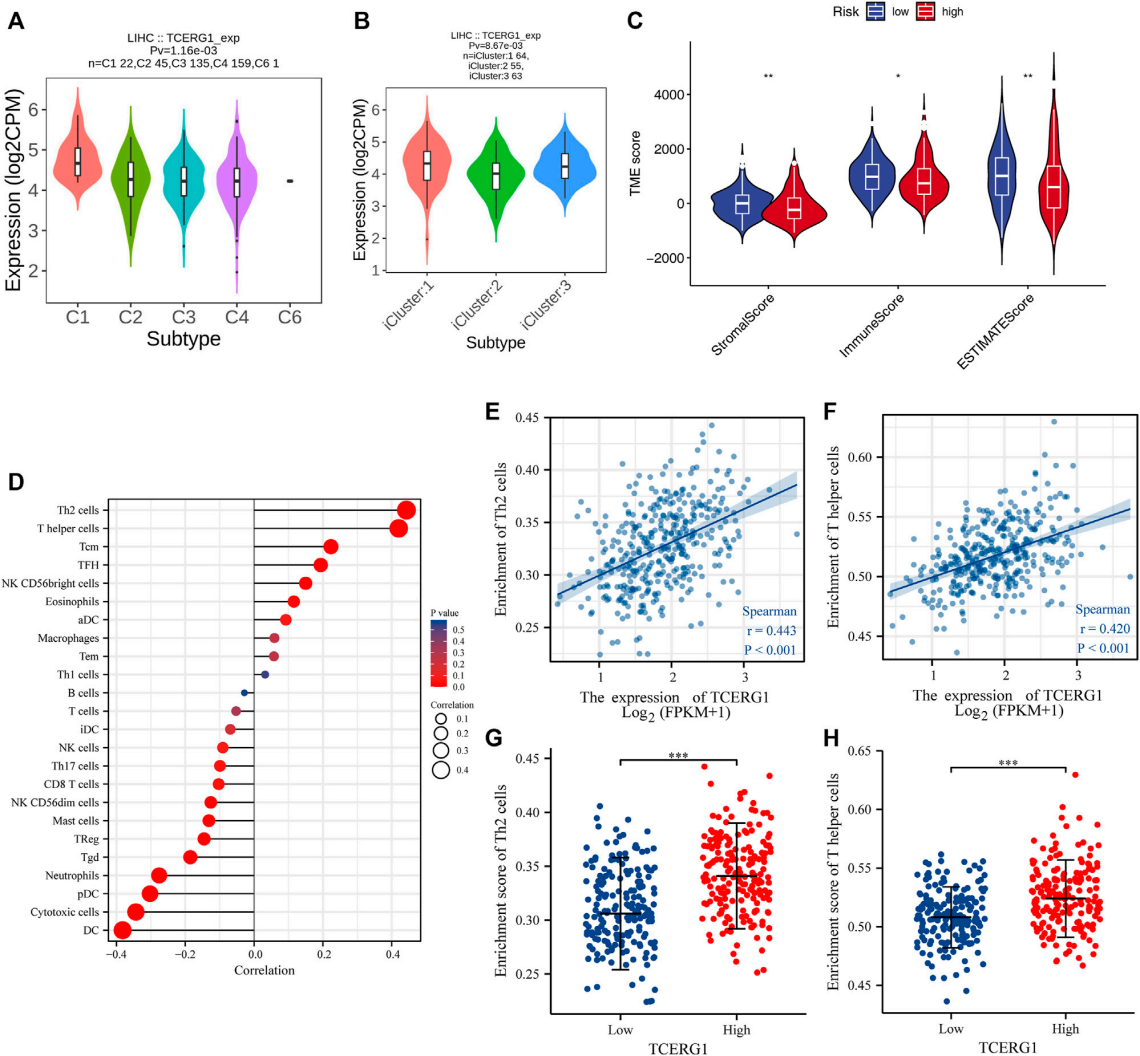
Correlation between *TCERG1* gene expression and immune infiltration of tumor microenvironment. **(A,B)** Correlation between *TCERG1* expression and immune, molecular subtypes of hepatocellular carcinoma; **(C)** Correlation between *TCERG1* score and immune, stromal scores; **(D)** Correlation between *TCERG1* and 24 immune cells using Spearman correlation method; **(E,F)** Correlation between *TCERG1* expression level and Th2 cells, T helper cells; **(G,H)** the correlation between *TCERG1* gene expression level and the abundance of Th2 cells, T helper cells using Wilcoxon rank sum test.

*TCERG1* was found to be significantly enriched in immune cells by GSEA enrichment analysis. Next, through the TISIDB website, we explored the role of *TCERG1* gene expression in the immune and molecular subtypes of hepatocellular carcinoma. The results showed that in HCC, *TCERG1* was highly expressed in C1 and C4. We also noticed that *TCERG1* expression in HCC was correlated with molecular subtypes, in which iCluster1 was high expression (Figures 9A,B).

After demonstrating the differential expression of *TCERG1* in different immune and molecular subtypes, we explored the potential relationship between *TCERG1* expression and immune cell infiltration in hepatocellular carcinoma. The results showed that the StromalScore, Immunescore, and EstimateScore were higher in the low-expressing HCC group (Figure 9C). Through spearman correlation analysis, the relationship between the expression level of *TCERG1* in the HCC tumor microenvironment and the level of immune cell infiltration by the ssGSEA quorum was determined. The results showed that the expression of *TCERG1* was positively correlated with Th2 cells, T helper cells, Tcm, TFH, NK CD56 bright cells, Eosinophils; and negatively correlated with DCs, Cytotoxic cells, pDCs, Mast cells, Neutrophils, Tgd, and TReg (Figure 9D). Among them, Th2 cells and T herper cells were significantly positively correlated with *TCERG1* expression, with Spearman R up to 0.443 and 0.420 with *p* values < 0.001. (Figures 9E–H). Therefore, our findings suggested that *TCERG1* was involved in regulating the functions of Th2 cells and T helper cells in HCC.

### Knockdown of *TCERG1* inhibits HCC cell proliferation, migration and invasion

**FIGURE 10:**
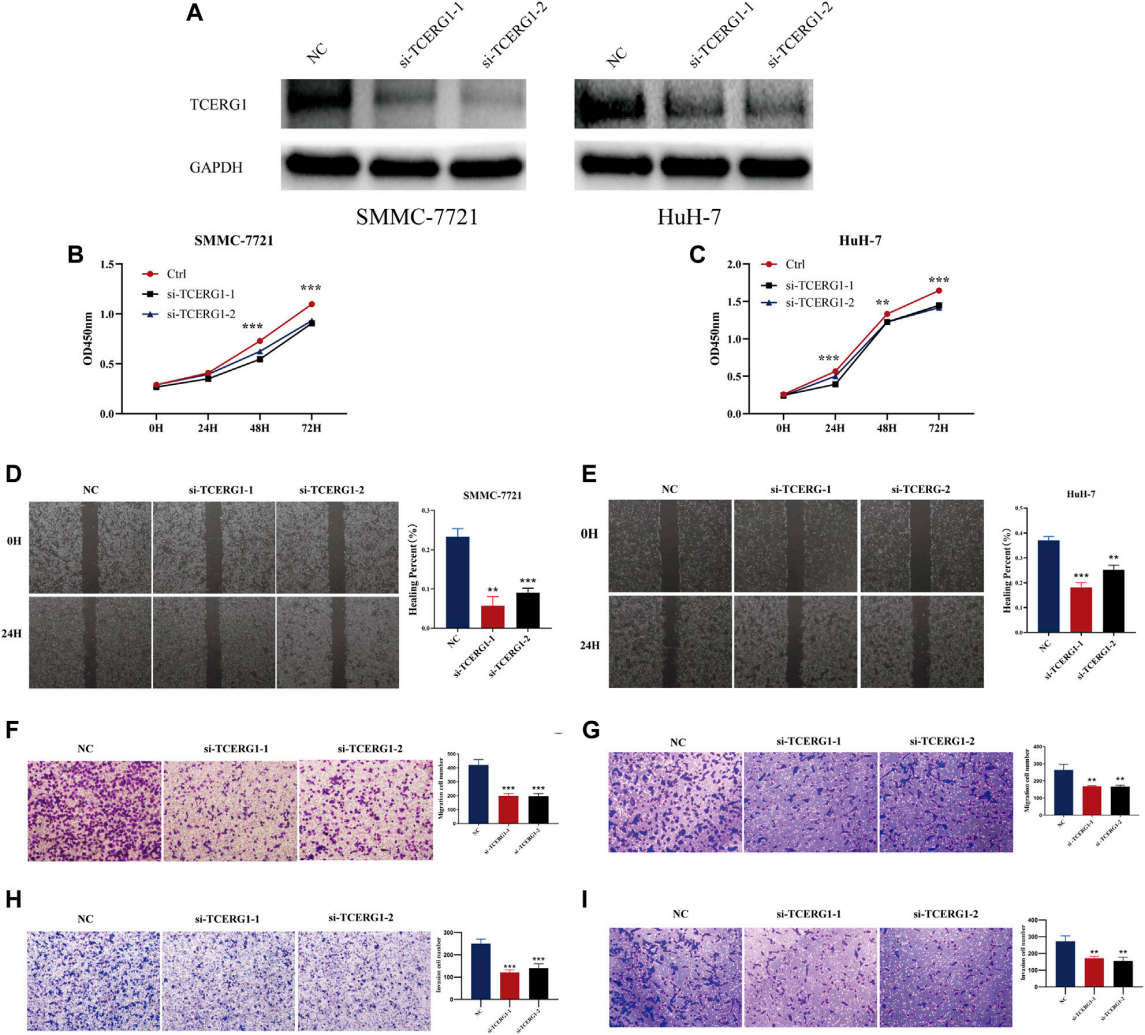
Knockdown of *TCERG1* in HCC cell suppresses proliferation, migration and invasion of HCC cells. **(A)** WB shows *TCERG1* protein levels under two different *TCERG1-siRNA* treatments, **(B,C)** cck8 assay on the proliferation ability of cells in NC and *Si-TCERG* groups. **(D,E)** Wound-healing assay for detection of cell migration in NC and *Si-TCERG1* cells. **(F,I)** Transwell assays for the effects of *TCERG1* knockdown on HCC cell migration and invasion. (Left: HCC cell line SMMC-7721, Right: HCC cell line Huh-7, quantitative analyses, n = 3, *p < 0.05; **p < 0.01; ***p < 0.001)

The results of RT-qPCR showed that *TCERG1* was highly expressed in hepatocellular carcinoma cell lines SNU-449, SMMC-7721, Huh-7 and MHCC97-H compared with normal hepatocytes LO2, and the difference was statistically significant. To investigate the biological function of *TCERG1* in HCC, 2 cell lines, SMMC-7721 and Huh-7, were selected for the experiment. Subsequently, *TCERG1* expression was reduced in HCC cell lines using two independent small interfering RNAs (siRNAs) and the efficiency of siRNA was verified by Western blot (Figure 10A); CCK8 results showed that knockdown of *TCERG1* inhibited the value-added viability of SMMC-7721 and Huh-7 cell lines compared to the NC group (Figures 10B,C).

The results of the scratch assay showed that the 24 h scratch width was wider in the *Si-TCERG1* group compared with the NC group, and knockdown of *TCERG1* decreased the wound healing ability of Huh-7, SMMC-7721 cells (Figures 10D,E). Transwell results showed that the number of migrating and invading cells crossing the chamber membrane at 24 h was significantly reduced in the *Si-TCERG1-1* group compared with the NC group (Figures 10F–I), indicating that knockdown of *TCERG1* significantly inhibited the migration and invasion of HCC cells.

## Discussion

HCC is a common type of cancer, and in recent years, its incidence has continued to increase with poor prognosis and high mortality (Zhao et al., 2020). Late diagnosis and rapid metastasis are the main causes of malignant death in patients with hepatocellular carcinoma. Although the study of hepatocellular carcinoma pathology has gradually advanced over the past few years and progress has been made in the diagnosis and management of treatment strategies for patients with hepatocellular carcinoma, but the specific molecular mechanisms of HCC metastasis are still unclear, the success rate and clinical outcomes of treatment remain poor (Lurje et al., 2019). The occurrence, development, and metastasis of liver cancer is a very complex process, and studies in recent years have shown that the occurrence and poor prognosis of HCC may be related to abnormal gene expression (Montes et al., 2012). With the continuous discovery of molecular biomarkers, prognostic indicators will become a promising clinical tool for liver cancer (Elliott and Sutterwala, 2015). It is necessary to find effective molecular mechanisms for the occurrence and development of HCC and to develop new biomarkers for liver cancer diagnosis and prognosis to aid in clinical diagnosis and prediction [39, 40].

TCERG1 is a highly conserved human protein, which plays an important role in regulating the transcription rate of RNA polymerase II and in alternative splicing. Studies have shown that *TCERG1* can stimulate the elongation of RNAP II, and its overexpression causes a shift to *Bcl-x*_*s*_ proapoptotic splice variant, which is involved in the regulation of apoptosis (Montes et al., 2012). The expression of *TCERG1* gene and the potential mechanisms of its prognostic impact on HCC patients have not been evaluated yet.

We used TCGA-LIHC data to assess the prognostic value of *TCERG1*. Our analysis is the first to identify *TCERG1* as a biomarker for hepatocellular carcinoma. In this study, the expression of *TCERG1* in pan-cancer was explored through multiple platforms, and further verified using qRT-PCR, and the results showed that the expression of *TCERG1* gene in tumor tissue was increased. By exploring the correlation between the *TCERG1* gene and clinical features, *TCERG1* overexpression was significantly associated with various clinical traits. Survival analysis found that patients with low expression of the *TCERG1* gene had better survival and high expression had significantly lower survival, and the results were validated in both the Kaplan Meier and GEPIA databases. Our study found that high expression of the *TCERG1* gene is associated with poor prognosis as an independent prognostic factor for OS in HCC patients. ROC analysis also validated this diagnostic value (AUC = 0.716). As well, these results are verified in an independent GEO cohort. However, its specific significance in the prognosis of HCC is unclear. In addition, *in vitro* experiments using *TCERG1* gene intervention in hepatocellular carcinoma cell lines SMMC-7721 and Huh-7 demonstrated that knockdown of *TCERG1* inhibited the proliferation, migration and invasion process of hepatocellular carcinoma cells, which is an important complement to the functional and clinical significance of *TCERG1*.

We next investigated the genes co-expressed with *TCERG1* using Linkedomics and CCLE databases. By taking the intersection of the top 50 genes positively associated with *TCERG1* in both databases, we found that *CPSF6* and *MAML1* may be involved in the development of hepatocellular carcinoma together with *TCERG1*. It has been reported that *CPSF6* (Tan et al., 2021) and *MAML1* (Wang et al., 2016) are highly expressed in HCC, and their overexpression is associated with poor prognosis of patients, and they promote the proliferation, migration and invasion of HCC cells *in vitro* and *in vivo*. The results of GO and KEGG further suggest that *TCERG1*, *CPSF6* and *MAML1* may be involved in the development of HCC through cell cycle, DNA replication and other physiological processes.

In addition, GSEA enrichment results showed that the high expression phenotype of *TCERG1* gene was closely associated with cell cycle, apoptosis, pathway in cancer and other signaling pathways. Apoptosis is a programmed form of cell death in multicellular organisms. Several studies have also found (Montes et al., 2015) that *TCERG1* affects both intrinsic and extrinsic apoptotic pathways, and *TCERG*1 sensitizes apoptotic agents and promotes apoptosis by modulating selective splicing of the key apoptotic genes *Bcl-x* and *Fas/CD95*. The specific mechanism for *TCERG1* in hepatocellular carcinoma apoptosis has not been reported in detail and needs to be further explored.

In GSEA enrichment results, we found that the high *TCERG1* phenotype was also enriched in immune cell-related signaling pathways such as B cell and T cell. Stromal Score, Immune score and Estimate Score were found to be higher in the low expression HCC group in TME. Next, we used ssGSEA and Spearman correlation analysis to explore the relationship between high *TCERG1* expression and immune infiltration level in hepatocellular carcinoma. We found that *TCERG1* expression was positively correlated with most immune cells, especially with Th2 cells, T herper cells. Th2 cells Elliott and Sutterwala (Elliott and Sutterwala, 2015) can produce Th2 cytokines, such as IL-4, IL-5,IL-10 and IL-13, which are important components of immune cells. Elevation of *TCERG1* expression increased Th2 cells and T herper cells. The findings may suggest that *TCERG1* has a function in regulating Th2 cells and T herper cells in HCC, but the exact mechanism is unclear and needs to be further explored.

In summary, our study suggest that *TCERG1* is upregulated in HCC and high *TCERG1* expression is associated with clinical progression and is considered to be an independent risk factor for OS in HCC patients. Co-expressed genes *CPSF6* and *MAMAL1* may be jointly involved in the development of hepatocellular carcinoma with *TCERG1*. In addition, cell cycle, cancer pathway, and Wnt signaling pathway may be key signaling pathways regulated by *TCERG1* in HCC. With a better understanding of its function, *TCERG1* could serve as an effective tool for the diagnosis and treatment of HCC and may help to make biomarker therapy a promising option for the treatment of liver disease in the future. However, the specific molecular mechanisms underlying the disease progression of HCC need to be further explored.

## Data Availability

The original contributions presented in the study are included in the article/Supplementary Material, further inquiries can be directed to the corresponding authors.
