# Supplementary material for: Overexpression of TCERG1 as a prognostic marker in hepatocellular carcinoma: A TCGA data-based analysis
Source: Front Genet. 2022 Oct 10;13:959832. doi: 10.3389/fgene.2022.959832 (PMC9589486; doi:10.3389/fgene.2022.959832)
Supplement: Supplementary file 1 [file DataSheet1.PDF]

Supplementary Table 1. *TCERG1* gene expression

ID	TCERG1	Type
TCGA-BD-A3EP-11A-12R-A22L-07	1.325703	Normal
TCGA-G3-A3CH-11A-11R-A22L-07	1.068857	Normal
TCGA-BC-A10X-11A-11R-A131-07	1.098255	Normal
TCGA-DD-A1EH-11A-11R-A131-07	0.896195	Normal
TCGA-DD-A11A-11A-11R-A131-07	1.345946	Normal
TCGA-DD-A3A6-11A-11R-A22L-07	0.313125	Normal
TCGA-DD-A1EB-11A-11R-A131-07	1.035474	Normal
TCGA-BC-A110-11A-11R-A131-07	1.711073	Normal
TCGA-BC-A216-11A-11R-A155-07	0.851022	Normal
TCGA-FV-A3R2-11A-11R-A22L-07	1.160674	Normal
TCGA-DD-A3A4-11A-11R-A22L-07	1.610387	Normal
TCGA-BC-A10Y-11A-11R-A131-07	1.469802	Normal
TCGA-BC-A10W-11A-11R-A131-07	1.313009	Normal
TCGA-DD-A119-11A-11R-A131-07	0.942361	Normal
TCGA-DD-A1EC-11A-11R-A131-07	1.295476	Normal
TCGA-DD-A1EJ-11A-11R-A155-07	1.259695	Normal
TCGA-BC-A10R-11A-11R-A131-07	1.455286	Normal
TCGA-FV-A3I1-11A-11R-A22L-07	1.028068	Normal
TCGA-BC-A10Q-11A-11R-A131-07	1.233852	Normal
TCGA-FV-A23B-11A-11R-A16W-07	1.402711	Normal
TCGA-BC-A10Z-11A-11R-A131-07	2.185204	Normal
TCGA-DD-A1EG-11A-11R-A213-07	1.205166	Normal
TCGA-DD-A11D-11A-12R-A131-07	1.249444	Normal
TCGA-BC-A10T-11A-11R-A131-07	1.194365	Normal
TCGA-ES-A2HT-11A-11R-A180-07	1.68978	Normal
TCGA-EP-A3RK-11A-11R-A22L-07	0.720607	Normal
TCGA-FV-A2QR-11A-11R-A213-07	1.516604	Normal
TCGA-DD-A11C-11A-11R-A131-07	1.277818	Normal
TCGA-DD-A39W-11A-11R-A213-07	0.626233	Normal
TCGA-DD-A3A1-11A-11R-A213-07	0.381096	Normal
TCGA-DD-A113-11A-12R-A131-07	0.982045	Normal
TCGA-DD-A39X-11A-11R-A213-07	0.603798	Normal
TCGA-DD-A3A5-11A-11R-A22L-07	0.643975	Normal
TCGA-DD-A3A2-11A-11R-A213-07	0.671608	Normal
TCGA-EP-A12J-11A-11R-A131-07	1.042878	Normal
TCGA-BC-A10U-11A-11R-A131-07	1.410028	Normal
TCGA-DD-A118-11A-11R-A131-07	0.870642	Normal
TCGA-DD-A1EL-11A-11R-A155-07	1.091785	Normal
TCGA-DD-A3A3-11A-11R-A22L-07	1.003121	Normal
TCGA-DD-A116-11A-12R-A26B-07	0.90171	Normal
TCGA-DD-A1EI-11A-11R-A131-07	1.061091	Normal
TCGA-BD-A2L6-11A-21R-A213-07	2.031865	Normal
TCGA-FV-A3I0-11A-11R-A22L-07	1.151579	Normal
TCGA-DD-A1EE-11A-11R-A131-07	1.064623	Normal
TCGA-DD-A3A8-11A-11R-A22L-07	0.277253	Normal

TCGA-DD-A11B-11A-11R-A131-07	0.811952	Normal
TCGA-DD-A114-11A-12R-A131-07	1.364469	Normal
TCGA-DD-A39Z-11A-21R-A213-07	0.96457	Normal
TCGA-EP-A26S-11A-12R-A16W-07	0.48539	Normal
TCGA-DD-A39V-11A-11R-A213-07	0.784253	Normal
TCGA-DD-AAVS-01A-11R-A41C-07	3.889933	Tumor
TCGA-DD-AAE3-01A-11R-A41C-07	1.031703	Tumor
TCGA-DD-A4NS-01A-11R-A311-07	3.814761	Tumor
TCGA-5R-AA1D-01A-11R-A38B-07	2.255798	Tumor
TCGA-BW-A5NO-01A-11R-A27V-07	1.745618	Tumor
TCGA-DD-AADL-01A-11R-A41C-07	2.877878	Tumor
TCGA-XR-A8TC-01A-11R-A36F-07	5.581745	Tumor
TCGA-2Y-A9GS-01A-12R-A38B-07	4.075525	Tumor
TCGA-CC-A1HT-01A-11R-A131-07	7.006956	Tumor
TCGA-DD-AACL-01A-11R-A41C-07	4.208516	Tumor
TCGA-ED-A7XP-01A-11R-A352-07	5.410586	Tumor
TCGA-G3-AAUZ-01A-11R-A38B-07	0.800426	Tumor
TCGA-DD-A3A5-01A-11R-A22L-07	1.081847	Tumor
TCGA-RC-A7SK-01A-11R-A352-07	1.988441	Tumor
TCGA-DD-A73D-01A-12R-A32O-07	2.149189	Tumor
TCGA-RC-A6M5-01A-11R-A32O-07	1.80344	Tumor
TCGA-G3-AAV4-01A-11R-A38B-07	2.187451	Tumor
TCGA-2Y-A9H1-01A-11R-A38B-07	0.866991	Tumor
TCGA-DD-AACI-01A-11R-A41C-07	1.270426	Tumor
TCGA-DD-AAVP-01A-11R-A41C-07	1.847123	Tumor
TCGA-EP-A26S-01A-11R-A16W-07	2.364445	Tumor
TCGA-DD-A4NB-01A-12R-A266-07	2.090935	Tumor
TCGA-LG-A6GG-01A-11R-A311-07	3.330223	Tumor
TCGA-4R-AA8I-01A-11R-A38B-07	2.744849	Tumor
TCGA-ED-A5KG-01A-11R-A27V-07	5.43714	Tumor
TCGA-DD-AADO-01A-11R-A41C-07	0.993292	Tumor
TCGA-DD-AADK-01A-11R-A41C-07	2.156582	Tumor
TCGA-LG-A9QD-01A-11R-A38B-07	2.206589	Tumor
TCGA-EP-A3RK-01A-11R-A22L-07	2.308028	Tumor
TCGA-DD-AACA-02A-11R-A41C-07	3.134415	Tumor
TCGA-2Y-A9H3-01A-11R-A38B-07	1.39981	Tumor
TCGA-BW-A5NQ-01A-11R-A27V-07	5.050528	Tumor
TCGA-CC-A5UE-01A-11R-A28V-07	3.090277	Tumor
TCGA-CC-5259-01A-31R-A213-07	0.621668	Tumor
TCGA-FV-A23B-01A-11R-A16W-07	4.285524	Tumor
TCGA-ZP-A9CV-01A-11R-A38B-07	2.235991	Tumor
TCGA-ES-A2HS-01A-11R-A180-07	0.508273	Tumor
TCGA-2Y-A9H8-01A-11R-A39D-07	2.366502	Tumor
TCGA-G3-A5SK-01A-11R-A27V-07	1.267851	Tumor
TCGA-DD-AACO-01A-11R-A41C-07	1.123882	Tumor
TCGA-DD-AAW3-01A-11R-A41C-07	2.608931	Tumor
TCGA-ED-A4XI-01A-11R-A266-07	1.776488	Tumor
TCGA-ZP-A9D2-01A-11R-A38B-07	3.20065	Tumor
TCGA-G3-AAV3-01A-11R-A37K-07	1.764271	Tumor
TCGA-K7-AAU7-01A-11R-A38B-07	6.653045	Tumor

TCGA-MI-A75I-01A-11R-A32O-07	2.616832	Tumor
TCGA-CC-5263-01A-01R-A131-07	6.712341	Tumor
TCGA-DD-A3A9-01A-11R-A266-07	1.483426	Tumor
TCGA-G3-A5SM-01A-12R-A28V-07	1.937718	Tumor
TCGA-DD-AADV-01A-11R-A39D-07	2.618582	Tumor
TCGA-DD-A11B-01A-11R-A131-07	2.137216	Tumor
TCGA-DD-A11A-01A-11R-A131-07	1.71781	Tumor
TCGA-ZP-A9D0-01A-11R-A37K-07	1.738035	Tumor
TCGA-RC-A6M6-01A-11R-A32O-07	4.759276	Tumor
TCGA-DD-A4NN-01A-11R-A28V-07	3.717496	Tumor
TCGA-DD-AACG-01A-11R-A41C-07	1.65018	Tumor
TCGA-UB-A7MC-01A-11R-A33R-07	5.071285	Tumor
TCGA-DD-A1EA-01A-11R-A131-07	3.605263	Tumor
TCGA-DD-A4NG-01A-11R-A27V-07	3.397439	Tumor
TCGA-O8-A75V-01A-11R-A32O-07	1.549065	Tumor
TCGA-DD-A39V-01A-11R-A213-07	1.141963	Tumor
TCGA-BC-A69I-01A-11R-A311-07	1.194435	Tumor
TCGA-ZS-A9CF-02A-11R-A38B-07	3.902278	Tumor
TCGA-DD-A4ND-01A-11R-A266-07	5.522463	Tumor
TCGA-DD-AADC-01A-11R-A41C-07	9.230503	Tumor
TCGA-DD-AAEH-01A-11R-A41C-07	1.739163	Tumor
TCGA-ZP-A9D1-01A-11R-A38B-07	4.360256	Tumor
TCGA-BC-A217-01A-11R-A155-07	3.185991	Tumor
TCGA-DD-AAE9-01A-11R-A41C-07	1.53919	Tumor
TCGA-G3-A25S-01A-11R-A16W-07	2.125075	Tumor
TCGA-BC-A10S-01A-22R-A131-07	1.153461	Tumor
TCGA-NI-A4U2-01A-11R-A28V-07	1.493687	Tumor
TCGA-G3-A3CK-01A-11R-A213-07	1.765925	Tumor
TCGA-RC-A7SF-01A-11R-A352-07	3.105286	Tumor
TCGA-DD-AACD-01A-11R-A41C-07	1.243669	Tumor
TCGA-2Y-A9H0-01A-11R-A38B-07	2.774777	Tumor
TCGA-DD-A3A6-01A-11R-A22L-07	1.758227	Tumor
TCGA-DD-AAW2-01A-11R-A41C-07	2.662271	Tumor
TCGA-3K-AAZ8-01A-12R-A39D-07	2.014229	Tumor
TCGA-DD-A4NH-01A-11R-A27V-07	5.714012	Tumor
TCGA-MI-A75H-01A-11R-A32O-07	1.406113	Tumor
TCGA-DD-AAEB-01A-11R-A41C-07	1.159969	Tumor
TCGA-ZP-A9CY-01A-11R-A38B-07	1.675371	Tumor
TCGA-BD-A3EP-01A-11R-A22L-07	2.983885	Tumor
TCGA-EP-A2KB-01A-11R-A180-07	3.529212	Tumor
TCGA-CC-A8HU-01A-11R-A36F-07	3.805117	Tumor
TCGA-DD-AADF-01A-11R-A41C-07	1.836277	Tumor
TCGA-DD-AACH-01A-11R-A41C-07	5.940702	Tumor
TCGA-BC-A10X-01A-11R-A131-07	1.513176	Tumor
TCGA-CC-5262-01A-01R-A131-07	1.488353	Tumor
TCGA-CC-A7IK-01A-12R-A33R-07	3.685979	Tumor
TCGA-RC-A7SH-01A-11R-A38B-07	5.06634	Tumor
TCGA-ED-A7XO-01A-11R-A352-07	1.93372	Tumor
TCGA-G3-A7M6-01A-11R-A33R-07	3.75466	Tumor
TCGA-G3-A7M8-01A-11R-A33R-07	1.17644	Tumor

TCGA-DD-AAW1-01A-11R-A41C-07	1.385206	Tumor
TCGA-G3-A7M7-01A-12R-A352-07	1.519766	Tumor
TCGA-ED-A7PZ-01A-11R-A33R-07	1.709304	Tumor
TCGA-CC-A7IG-01A-11R-A33J-07	2.612756	Tumor
TCGA-CC-5261-01A-01R-A131-07	4.614204	Tumor
TCGA-WQ-A9G7-01A-11R-A37K-07	5.915218	Tumor
TCGA-WX-AA47-01A-11R-A39D-07	2.229027	Tumor
TCGA-ZP-A9CZ-01A-11R-A38B-07	2.972544	Tumor
TCGA-DD-AA3A-01A-11R-A37K-07	12.32322	Tumor
TCGA-DD-A3A4-01A-11R-A22L-07	0.681739	Tumor
TCGA-DD-A39Y-01A-11R-A213-07	3.471551	Tumor
TCGA-2Y-A9H5-01A-11R-A38B-07	3.418743	Tumor
TCGA-DD-A114-01A-11R-A131-07	4.432992	Tumor
TCGA-DD-A4NI-01A-11R-A27V-07	1.775575	Tumor
TCGA-BC-A110-01A-11R-A131-07	1.45841	Tumor
TCGA-UB-A7MA-01A-11R-A33R-07	3.956784	Tumor
TCGA-CC-A3MB-01A-11R-A213-07	4.900546	Tumor
TCGA-KR-A7K2-01A-12R-A33R-07	2.509582	Tumor
TCGA-DD-AACK-01A-11R-A41C-07	3.023071	Tumor
TCGA-FV-A3I0-01A-11R-A22L-07	3.404305	Tumor
TCGA-RC-A7SB-01A-21R-A352-07	1.281826	Tumor
TCGA-GJ-A9DB-01A-11R-A37K-07	3.563317	Tumor
TCGA-UB-A7ME-01A-11R-A33J-07	2.308415	Tumor
TCGA-DD-AADU-01A-11R-A41C-07	2.183041	Tumor
TCGA-2Y-A9HB-01A-11R-A39D-07	2.405164	Tumor
TCGA-CC-A9FV-01A-11R-A37K-07	2.846523	Tumor
TCGA-DD-A1EK-01A-11R-A213-07	1.591892	Tumor
TCGA-DD-AAE1-01A-11R-A41C-07	3.692177	Tumor
TCGA-BC-A10U-01A-11R-A131-07	2.182629	Tumor
TCGA-DD-AACV-01A-11R-A41C-07	3.187444	Tumor
TCGA-DD-AAVY-01A-11R-A41C-07	2.663416	Tumor
TCGA-FV-A3R3-01A-11R-A22L-07	1.696557	Tumor
TCGA-ED-A97K-01A-21R-A38B-07	6.579481	Tumor
TCGA-BC-A10W-01A-11R-A131-07	5.316071	Tumor
TCGA-DD-A3A8-01A-11R-A22L-07	1.628105	Tumor
TCGA-DD-AACT-01A-11R-A41C-07	1.801063	Tumor
TCGA-5R-AA1C-01A-11R-A41C-07	1.87146	Tumor
TCGA-5R-AAAM-01A-12R-A41C-07	1.606206	Tumor
TCGA-BW-A5NP-01A-11R-A27V-07	5.344786	Tumor
TCGA-WX-AA44-01A-11R-A39D-07	3.897137	Tumor
TCGA-DD-A1EH-01A-11R-A131-07	3.905691	Tumor
TCGA-DD-A3A1-01A-11R-A213-07	1.611495	Tumor
TCGA-KR-A7K0-01A-12R-A33R-07	2.813568	Tumor
TCGA-FV-A3R2-01A-11R-A22L-07	3.195721	Tumor
TCGA-DD-AACN-01A-11R-A41C-07	5.309395	Tumor
TCGA-DD-AAE4-01A-11R-A41C-07	0.68021	Tumor
TCGA-DD-AAEG-01A-11R-A39D-07	2.735822	Tumor
TCGA-DD-A1EJ-01A-11R-A155-07	3.481636	Tumor
TCGA-DD-A4NR-01A-11R-A311-07	5.238731	Tumor
TCGA-FV-A2QQ-01A-11R-A22L-07	2.211122	Tumor

TCGA-G3-AAV7-01A-11R-A38B-07	4.0925	Tumor
TCGA-CC-A9FW-01A-11R-A37K-07	3.322485	Tumor
TCGA-RC-A6M4-01A-11R-A32O-07	1.408988	Tumor
TCGA-G3-A3CG-01A-11R-A213-07	2.896597	Tumor
TCGA-DD-A11C-01A-11R-A131-07	2.561324	Tumor
TCGA-DD-AAD2-01A-11R-A41C-07	2.610339	Tumor
TCGA-NI-A8LF-01A-11R-A36F-07	2.050229	Tumor
TCGA-DD-AADN-01A-11R-A41C-07	1.120176	Tumor
TCGA-EP-A12J-01A-11R-A131-07	1.741932	Tumor
TCGA-DD-A73G-01A-22R-A32O-07	0.656844	Tumor
TCGA-5C-AAPD-01A-21R-A39D-07	1.501355	Tumor
TCGA-DD-AAED-01A-12R-A41C-07	6.589223	Tumor
TCGA-DD-AADJ-01A-11R-A41C-07	1.51553	Tumor
TCGA-DD-AAVZ-01A-11R-A41C-07	3.194848	Tumor
TCGA-XR-A8TF-01A-11R-A36F-07	5.923985	Tumor
TCGA-DD-AACF-01A-11R-A41C-07	2.261418	Tumor
TCGA-FV-A495-01A-11R-A266-07	0.928779	Tumor
TCGA-DD-A3A3-01A-11R-A22L-07	1.086341	Tumor
TCGA-MI-A75C-01A-11R-A32O-07	2.311569	Tumor
TCGA-DD-AAVX-01A-11R-A41C-07	2.428539	Tumor
TCGA-DD-AAE7-01A-11R-A41C-07	1.243229	Tumor
TCGA-ZS-A9CE-01A-11R-A37K-07	1.68195	Tumor
TCGA-CC-5264-01A-01R-A131-07	3.907099	Tumor
TCGA-HP-A5N0-01A-11R-A28V-07	1.600013	Tumor
TCGA-G3-A5SJ-01A-11R-A27V-07	3.315598	Tumor
TCGA-DD-A3A2-01A-11R-A213-07	0.798866	Tumor
TCGA-2Y-A9H6-01A-11R-A39D-07	2.861009	Tumor
TCGA-DD-AAEE-01A-11R-A41C-07	3.504285	Tumor
TCGA-UB-AA0U-01A-11R-A38B-07	3.822807	Tumor
TCGA-CC-A3M9-01A-11R-A213-07	4.886853	Tumor
TCGA-CC-A8HT-01A-11R-A36F-07	5.778373	Tumor
TCGA-WQ-AB4B-01A-11R-A41C-07	1.915491	Tumor
TCGA-DD-AADY-01A-11R-A41C-07	3.58499	Tumor
TCGA-2Y-A9GZ-01A-11R-A39D-07	2.055275	Tumor
TCGA-DD-A11D-01A-11R-A131-07	1.956147	Tumor
TCGA-KR-A7K7-01A-11R-A33J-07	2.207876	Tumor
TCGA-BC-4072-01B-11R-A155-07	3.08337	Tumor
TCGA-K7-A5RF-01A-11R-A28V-07	1.08871	Tumor
TCGA-DD-AAVU-01A-11R-A41C-07	1.177311	Tumor
TCGA-DD-A73B-01A-12R-A32O-07	2.479593	Tumor
TCGA-PD-A5DF-01A-11R-A27V-07	2.969756	Tumor
TCGA-BC-A8YO-01A-11R-A37K-07	5.817141	Tumor
TCGA-EP-A2KA-01A-11R-A180-07	2.386675	Tumor
TCGA-DD-AACQ-01A-11R-A41C-07	2.266273	Tumor
TCGA-DD-AADI-01A-11R-A41C-07	2.782757	Tumor
TCGA-CC-A5UD-01A-11R-A28V-07	3.421344	Tumor
TCGA-ZS-A9CD-01A-11R-A37K-07	1.920057	Tumor
TCGA-DD-A1ED-01A-11R-A155-07	1.129982	Tumor
TCGA-DD-AAEK-01A-11R-A41C-07	2.687526	Tumor
TCGA-BC-A10T-01A-11R-A131-07	2.618083	Tumor

TCGA-DD-AAC8-01A-11R-A41C-07	4.298436	Tumor
TCGA-XR-A8TE-01A-11R-A36F-07	3.829236	Tumor
TCGA-K7-A6G5-01A-11R-A311-07	1.121	Tumor
TCGA-WX-AA46-01A-11R-A39D-07	1.639146	Tumor
TCGA-DD-AADD-01A-11R-A41C-07	3.617199	Tumor
TCGA-DD-A1EF-01A-11R-A131-07	3.96388	Tumor
TCGA-DD-AACP-01A-11R-A41C-07	5.2612	Tumor
TCGA-RC-A6M3-01A-11R-A32O-07	7.31526	Tumor
TCGA-G3-A3CJ-01A-11R-A213-07	0.56609	Tumor
TCGA-DD-AAVR-01A-11R-A41C-07	2.316428	Tumor
TCGA-DD-AAVQ-01A-11R-A41C-07	2.552799	Tumor
TCGA-G3-A3CI-01A-11R-A213-07	1.089302	Tumor
TCGA-ED-A8O5-01A-11R-A36F-07	4.266772	Tumor
TCGA-BC-A5W4-01A-11R-A28V-07	1.538199	Tumor
TCGA-DD-AAD6-01A-11R-A41C-07	5.666527	Tumor
TCGA-K7-A5RG-01A-11R-A28V-07	4.37566	Tumor
TCGA-G3-AAV5-01A-11R-A37K-07	2.186795	Tumor
TCGA-2Y-A9GY-01A-11R-A38B-07	2.854759	Tumor
TCGA-UB-A7MD-01A-12R-A352-07	1.932435	Tumor
TCGA-DD-A4NF-01A-11R-A27V-07	1.942644	Tumor
TCGA-DD-A113-01A-11R-A131-07	2.940765	Tumor
TCGA-DD-AAW0-01A-11R-A41C-07	3.661845	Tumor
TCGA-DD-A1EI-01A-11R-A131-07	3.263441	Tumor
TCGA-CC-A7IF-01A-11R-A33J-07	2.764147	Tumor
TCGA-2Y-A9GW-01A-11R-A38B-07	2.995531	Tumor
TCGA-2Y-A9H4-01A-11R-A38B-07	2.085053	Tumor
TCGA-BC-A3KF-01A-11R-A213-07	2.759334	Tumor
TCGA-DD-A39Z-01A-11R-A213-07	1.013165	Tumor
TCGA-CC-5260-01A-01R-A131-07	4.995359	Tumor
TCGA-DD-AAE0-01A-11R-A41C-07	3.157759	Tumor
TCGA-DD-A116-01A-11R-A131-07	1.00629	Tumor
TCGA-RC-A7S9-01A-11R-A33R-07	2.144073	Tumor
TCGA-FV-A496-01A-11R-A266-07	1.864874	Tumor
TCGA-BC-A3KG-01A-11R-A213-07	6.274865	Tumor
TCGA-DD-A1EG-01A-11R-A213-07	2.284833	Tumor
TCGA-DD-A1EL-01A-11R-A155-07	3.981678	Tumor
TCGA-2Y-A9GU-01A-11R-A38B-07	2.109233	Tumor
TCGA-G3-A25V-01A-11R-A16W-07	1.941079	Tumor
TCGA-ED-A66Y-01A-11R-A311-07	4.861879	Tumor
TCGA-DD-AADB-01A-11R-A41C-07	3.168724	Tumor
TCGA-YA-A8S7-01A-11R-A37K-07	4.346566	Tumor
TCGA-GJ-A3OU-01A-31R-A38B-07	3.326809	Tumor
TCGA-BD-A2L6-01A-11R-A213-07	1.571478	Tumor
TCGA-ED-A627-01A-12R-A311-07	2.310488	Tumor
TCGA-ED-A7PX-01A-51R-A352-07	4.6368	Tumor
TCGA-2Y-A9GX-01A-11R-A38B-07	3.570092	Tumor
TCGA-DD-A115-01A-11R-A131-07	1.780244	Tumor
TCGA-DD-A73C-01A-12R-A33J-07	1.699095	Tumor
TCGA-MI-A75G-01A-11R-A32O-07	1.502503	Tumor
TCGA-G3-AAV0-01A-11R-A37K-07	0.856524	Tumor

TCGA-CC-A8HS-01A-11R-A36F-07	7.372438	Tumor
TCGA-DD-AACY-01A-11R-A41C-07	1.786912	Tumor
TCGA-CC-A7IE-01A-21R-A38B-07	4.634447	Tumor
TCGA-2Y-A9HA-01A-11R-A39D-07	3.474672	Tumor
TCGA-G3-A6UC-01A-21R-A33J-07	1.993136	Tumor
TCGA-CC-A7IH-01A-11R-A33J-07	2.462384	Tumor
TCGA-ZP-A9D4-01A-11R-A37K-07	2.526157	Tumor
TCGA-MI-A75E-01A-11R-A32O-07	1.295961	Tumor
TCGA-DD-AAD5-01A-11R-A41C-07	5.276722	Tumor
TCGA-DD-A39W-01A-11R-A213-07	2.87863	Tumor
TCGA-2Y-A9GT-01A-11R-A38B-07	2.271045	Tumor
TCGA-ED-A8O6-01A-11R-A36F-07	3.064994	Tumor
TCGA-DD-A39X-01A-11R-A213-07	1.261004	Tumor
TCGA-2Y-A9GV-01A-11R-A38B-07	2.553406	Tumor
TCGA-DD-AACJ-01A-11R-A41C-07	1.464282	Tumor
TCGA-DD-AAE6-01A-11R-A41C-07	2.717134	Tumor
TCGA-5C-A9VG-01A-11R-A37K-07	6.27788	Tumor
TCGA-DD-AADS-01A-11R-A41C-07	0.340954	Tumor
TCGA-DD-A4NV-01A-11R-A311-07	2.530631	Tumor
TCGA-DD-AACC-01A-11R-A41C-07	2.429762	Tumor
TCGA-FV-A4ZQ-01A-11R-A266-07	5.547934	Tumor
TCGA-DD-A73E-01A-12R-A32O-07	2.103002	Tumor
TCGA-DD-AAEA-01A-11R-A41C-07	2.999257	Tumor
TCGA-HP-A5MZ-01A-21R-A27V-07	2.414207	Tumor
TCGA-MR-A8JO-01A-12R-A36F-07	3.554173	Tumor
TCGA-G3-A25T-01A-11R-A16W-07	3.324935	Tumor
TCGA-BC-A112-01A-11R-A131-07	6.258388	Tumor
TCGA-DD-A73A-01A-12R-A32O-07	3.141835	Tumor
TCGA-BC-4073-01B-02R-A131-07	4.336635	Tumor
TCGA-DD-A4NJ-01A-11R-A27V-07	5.339938	Tumor
TCGA-ED-A7PY-01A-11R-A33R-07	2.466072	Tumor
TCGA-DD-A4NQ-01A-21R-A28V-07	3.304315	Tumor
TCGA-G3-A25U-01A-11R-A16W-07	3.416931	Tumor
TCGA-BD-A3ER-01A-11R-A213-07	2.536291	Tumor
TCGA-BC-A10R-01A-11R-A131-07	1.560151	Tumor
TCGA-DD-AACS-01A-11R-A41C-07	3.200168	Tumor
TCGA-DD-AACA-02B-11R-A41C-07	1.565401	Tumor
TCGA-DD-A1EE-01A-11R-A131-07	3.657944	Tumor
TCGA-CC-A7IL-01A-11R-A33R-07	1.566801	Tumor
TCGA-CC-A7IJ-01A-11R-A33R-07	4.382538	Tumor
TCGA-DD-AADA-01A-11R-A41C-07	2.605247	Tumor
TCGA-FV-A4ZP-01A-12R-A266-07	0.370591	Tumor
TCGA-G3-A7M9-01A-23R-A352-07	6.392106	Tumor
TCGA-CC-A5UC-01A-11R-A28V-07	3.949194	Tumor
TCGA-EP-A3JL-01A-11R-A213-07	1.839899	Tumor
TCGA-G3-A25Y-01A-11R-A16W-07	5.403887	Tumor
TCGA-DD-AADQ-01A-11R-A41C-07	3.45909	Tumor
TCGA-5C-A9VH-01A-11R-A37K-07	3.995023	Tumor
TCGA-UB-AA0V-01A-11R-A38B-07	2.024979	Tumor
TCGA-DD-A4NE-01A-11R-A27V-07	3.425615	Tumor

TCGA-DD-A119-01A-11R-A131-07	1.669752	Tumor
TCGA-G3-A3CH-01A-11R-A22L-07	2.313397	Tumor
TCGA-DD-AACZ-01A-11R-A41C-07	4.529446	Tumor
TCGA-WJ-A86L-01A-12R-A39D-07	3.09119	Tumor
TCGA-CC-A9FS-01A-11R-A37K-07	3.45977	Tumor
TCGA-G3-AAV2-01A-11R-A37K-07	1.269508	Tumor
TCGA-LG-A9QC-01A-11R-A37K-07	3.490781	Tumor
TCGA-ED-A82E-01A-11R-A352-07	4.712989	Tumor
TCGA-DD-A4NK-01A-11R-A28V-07	3.434081	Tumor
TCGA-DD-AAD3-01A-11R-A41C-07	1.64317	Tumor
TCGA-DD-AACU-01A-11R-A41C-07	2.694456	Tumor
TCGA-KR-A7K8-01A-11R-A33J-07	1.992388	Tumor
TCGA-DD-AADM-01A-11R-A41C-07	1.646563	Tumor
TCGA-CC-A3MC-01A-11R-A22L-07	3.736085	Tumor
TCGA-G3-A5SL-01A-11R-A27V-07	2.121369	Tumor
TCGA-UB-A7MB-01A-11R-A33R-07	3.309545	Tumor
TCGA-DD-A1EB-01A-11R-A131-07	1.691751	Tumor
TCGA-BC-A10Z-01A-11R-A131-07	2.91762	Tumor
TCGA-BC-A216-01A-11R-A155-07	5.183955	Tumor
TCGA-BC-A69H-01A-11R-A311-07	1.414442	Tumor
TCGA-CC-A9FU-01A-11R-A37K-07	3.946883	Tumor
TCGA-G3-A7M5-01A-11R-A33R-07	1.437671	Tumor
TCGA-BC-A10Y-01A-11R-A131-07	2.707116	Tumor
TCGA-DD-AAVV-01A-11R-A41C-07	3.23579	Tumor
TCGA-DD-A4NP-01A-11R-A28V-07	1.01557	Tumor
TCGA-DD-AADR-01A-11R-A41C-07	2.441964	Tumor
TCGA-BC-A10Q-01A-11R-A131-07	4.092977	Tumor
TCGA-CC-A8HV-01A-11R-A36F-07	6.144421	Tumor
TCGA-DD-AAEI-01A-11R-A41C-07	1.800363	Tumor
TCGA-CC-A3MA-01A-11R-A213-07	4.773913	Tumor
TCGA-FV-A3I1-01A-11R-A22L-07	2.950059	Tumor
TCGA-G3-AAV6-01A-21R-A37K-07	2.746125	Tumor
TCGA-CC-A123-01A-11R-A131-07	1.069429	Tumor
TCGA-G3-A5SI-01A-31R-A27V-07	2.743482	Tumor
TCGA-2Y-A9H2-01A-12R-A38B-07	6.000679	Tumor
TCGA-CC-A7II-01A-11R-A33J-07	5.084956	Tumor
TCGA-UB-A7MF-01A-11R-A33J-07	1.84407	Tumor
TCGA-DD-AAE2-01A-11R-A41C-07	2.074651	Tumor
TCGA-DD-A1EC-01A-21R-A131-07	3.030895	Tumor
TCGA-2V-A95S-01A-11R-A37K-07	3.251448	Tumor
TCGA-DD-AACE-01A-11R-A41C-07	1.833846	Tumor
TCGA-ED-A66X-01A-11R-A311-07	3.869055	Tumor
TCGA-DD-AACW-01A-11R-A41C-07	3.977482	Tumor
TCGA-DD-AAD0-01A-11R-A41C-07	2.664565	Tumor
TCGA-ZS-A9CF-01A-11R-A38B-07	2.82025	Tumor
TCGA-DD-A4NL-01A-11R-A28V-07	1.378173	Tumor
TCGA-DD-A4NA-01A-11R-A266-07	5.759716	Tumor
TCGA-T1-A6J8-01A-11R-A32O-07	1.945192	Tumor
TCGA-ED-A459-01A-11R-A266-07	3.056094	Tumor
TCGA-DD-AAD8-01A-11R-A41C-07	3.551132	Tumor

TCGA-XR-A8TD-01A-12R-A39D-07	2.833959	Tumor
TCGA-2Y-A9H7-01A-11R-A39D-07	2.832187	Tumor
TCGA-RG-A7D4-01A-12R-A33R-07	6.132205	Tumor
TCGA-ES-A2HT-01A-12R-A180-07	0.79721	Tumor
TCGA-QA-A7B7-01A-11R-A32O-07	4.142188	Tumor
TCGA-XR-A8TG-01A-11R-A36F-07	3.422749	Tumor
TCGA-DD-AACA-01A-11R-A41C-07	2.972509	Tumor
TCGA-DD-AAC9-01A-11R-A41C-07	1.021107	Tumor
TCGA-DD-A3A7-01A-11R-A22L-07	1.963898	Tumor
TCGA-MR-A520-01A-11R-A266-07	0.847233	Tumor
TCGA-ZS-A9CG-01A-11R-A37K-07	1.38597	Tumor
TCGA-EP-A2KC-01A-11R-A213-07	3.490314	Tumor
TCGA-DD-A73F-01A-11R-A32O-07	1.800382	Tumor
TCGA-DD-AADP-01A-11R-A39D-07	3.142852	Tumor
TCGA-GJ-A6C0-01A-12R-A311-07	2.677685	Tumor
TCGA-DD-AAVW-01A-11R-A41C-07	3.34731	Tumor
TCGA-2Y-A9H9-01A-21R-A39D-07	2.676858	Tumor
TCGA-G3-AAV1-01A-11R-A38B-07	3.889103	Tumor
TCGA-G3-A25X-01A-11R-A16W-07	1.669156	Tumor
TCGA-CC-5258-01A-01R-A131-07	4.812208	Tumor
TCGA-FV-A2QR-01A-11R-A213-07	2.022577	Tumor
TCGA-DD-A4NO-01A-11R-A28V-07	3.020786	Tumor
TCGA-DD-AAD1-01A-11R-A41C-07	4.456437	Tumor
TCGA-DD-AADW-01A-11R-A39D-07	4.297567	Tumor
TCGA-DD-AACB-01A-11R-A41C-07	1.761311	Tumor
TCGA-DD-A118-01A-11R-A131-07	1.952282	Tumor
TCGA-DD-AACX-01A-11R-A41C-07	2.24868	Tumor
TCGA-DD-AADG-01A-11R-A41C-07	2.054273	Tumor
TCGA-G3-A25Z-01A-11R-A16W-07	1.455661	Tumor

Supplementary Table 2. 50 pairs of normal and tumor samples from the same patient for the expression of the gene (*TCERG1*).

ID	TCERG1	Type
TCGA-BD-A3EP-11A-12R-A22L-07	1.325703	Normal
TCGA-G3-A3CH-11A-11R-A22L-07	1.068857	Normal
TCGA-BC-A10X-11A-11R-A131-07	1.098255	Normal
TCGA-DD-A1EH-11A-11R-A131-07	0.896195	Normal
TCGA-DD-A11A-11A-11R-A131-07	1.345946	Normal
TCGA-DD-A3A6-11A-11R-A22L-07	0.313125	Normal
TCGA-DD-A1EB-11A-11R-A131-07	1.035474	Normal
TCGA-BC-A110-11A-11R-A131-07	1.711073	Normal
TCGA-BC-A216-11A-11R-A155-07	0.851022	Normal
TCGA-FV-A3R2-11A-11R-A22L-07	1.160674	Normal
TCGA-DD-A3A4-11A-11R-A22L-07	1.610387	Normal
TCGA-BC-A10Y-11A-11R-A131-07	1.469802	Normal

TCGA-BC-A10W-11A-11R-A131-07	1.313009	Normal
TCGA-DD-A119-11A-11R-A131-07	0.942361	Normal
TCGA-DD-A1EC-11A-11R-A131-07	1.295476	Normal
TCGA-DD-A1EJ-11A-11R-A155-07	1.259695	Normal
TCGA-BC-A10R-11A-11R-A131-07	1.455286	Normal
TCGA-FV-A3I1-11A-11R-A22L-07	1.028068	Normal
TCGA-BC-A10Q-11A-11R-A131-07	1.233852	Normal
TCGA-FV-A23B-11A-11R-A16W-07	1.402711	Normal
TCGA-BC-A10Z-11A-11R-A131-07	2.185204	Normal
TCGA-DD-A1EG-11A-11R-A213-07	1.205166	Normal
TCGA-DD-A11D-11A-12R-A131-07	1.249444	Normal
TCGA-BC-A10T-11A-11R-A131-07	1.194365	Normal
TCGA-ES-A2HT-11A-11R-A180-07	1.68978	Normal
TCGA-EP-A3RK-11A-11R-A22L-07	0.720607	Normal
TCGA-FV-A2QR-11A-11R-A213-07	1.516604	Normal
TCGA-DD-A11C-11A-11R-A131-07	1.277818	Normal
TCGA-DD-A39W-11A-11R-A213-07	0.626233	Normal
TCGA-DD-A3A1-11A-11R-A213-07	0.381096	Normal
TCGA-DD-A113-11A-12R-A131-07	0.982045	Normal
TCGA-DD-A39X-11A-11R-A213-07	0.603798	Normal
TCGA-DD-A3A5-11A-11R-A22L-07	0.643975	Normal
TCGA-DD-A3A2-11A-11R-A213-07	0.671608	Normal
TCGA-EP-A12J-11A-11R-A131-07	1.042878	Normal
TCGA-BC-A10U-11A-11R-A131-07	1.410028	Normal
TCGA-DD-A118-11A-11R-A131-07	0.870642	Normal
TCGA-DD-A1EL-11A-11R-A155-07	1.091785	Normal
TCGA-DD-A3A3-11A-11R-A22L-07	1.003121	Normal
TCGA-DD-A116-11A-12R-A26B-07	0.90171	Normal
TCGA-DD-A1EI-11A-11R-A131-07	1.061091	Normal
TCGA-BD-A2L6-11A-21R-A213-07	2.031865	Normal
TCGA-FV-A3I0-11A-11R-A22L-07	1.151579	Normal
TCGA-DD-A1EE-11A-11R-A131-07	1.064623	Normal
TCGA-DD-A3A8-11A-11R-A22L-07	0.277253	Normal
TCGA-DD-A11B-11A-11R-A131-07	0.811952	Normal
TCGA-DD-A114-11A-12R-A131-07	1.364469	Normal
TCGA-DD-A39Z-11A-21R-A213-07	0.96457	Normal
TCGA-EP-A26S-11A-12R-A16W-07	0.48539	Normal
TCGA-DD-A39V-11A-11R-A213-07	0.784253	Normal
TCGA-DD-A3A5-01A-11R-A22L-07	1.081847	Tumor
TCGA-EP-A26S-01A-11R-A16W-07	2.364445	Tumor
TCGA-EP-A3RK-01A-11R-A22L-07	2.308028	Tumor
TCGA-FV-A23B-01A-11R-A16W-07	4.285524	Tumor
TCGA-DD-A11B-01A-11R-A131-07	2.137216	Tumor
TCGA-DD-A11A-01A-11R-A131-07	1.71781	Tumor
TCGA-DD-A39V-01A-11R-A213-07	1.141963	Tumor
TCGA-DD-A3A6-01A-11R-A22L-07	1.758227	Tumor
TCGA-BD-A3EP-01A-11R-A22L-07	2.983885	Tumor
TCGA-BC-A10X-01A-11R-A131-07	1.513176	Tumor
TCGA-DD-A3A4-01A-11R-A22L-07	0.681739	Tumor
TCGA-DD-A114-01A-11R-A131-07	4.432992	Tumor

TCGA-BC-A110-01A-11R-A131-07	1.45841	Tumor
TCGA-FV-A3I0-01A-11R-A22L-07	3.404305	Tumor
TCGA-BC-A10U-01A-11R-A131-07	2.182629	Tumor
TCGA-BC-A10W-01A-11R-A131-07	5.316071	Tumor
TCGA-DD-A3A8-01A-11R-A22L-07	1.628105	Tumor
TCGA-DD-A1EH-01A-11R-A131-07	3.905691	Tumor
TCGA-DD-A3A1-01A-11R-A213-07	1.611495	Tumor
TCGA-FV-A3R2-01A-11R-A22L-07	3.195721	Tumor
TCGA-DD-A1EJ-01A-11R-A155-07	3.481636	Tumor
TCGA-DD-A11C-01A-11R-A131-07	2.561324	Tumor
TCGA-EP-A12J-01A-11R-A131-07	1.741932	Tumor
TCGA-DD-A3A3-01A-11R-A22L-07	1.086341	Tumor
TCGA-DD-A3A2-01A-11R-A213-07	0.798866	Tumor
TCGA-DD-A11D-01A-11R-A131-07	1.956147	Tumor
TCGA-BC-A10T-01A-11R-A131-07	2.618083	Tumor
TCGA-DD-A113-01A-11R-A131-07	2.940765	Tumor
TCGA-DD-A1EI-01A-11R-A131-07	3.263441	Tumor
TCGA-DD-A39Z-01A-11R-A213-07	1.013165	Tumor
TCGA-DD-A116-01A-11R-A131-07	1.00629	Tumor
TCGA-DD-A1EG-01A-11R-A213-07	2.284833	Tumor
TCGA-DD-A1EL-01A-11R-A155-07	3.981678	Tumor
TCGA-BD-A2L6-01A-11R-A213-07	1.571478	Tumor
TCGA-DD-A39W-01A-11R-A213-07	2.87863	Tumor
TCGA-DD-A39X-01A-11R-A213-07	1.261004	Tumor
TCGA-BC-A10R-01A-11R-A131-07	1.560151	Tumor
TCGA-DD-A1EE-01A-11R-A131-07	3.657944	Tumor
TCGA-DD-A119-01A-11R-A131-07	1.669752	Tumor
TCGA-G3-A3CH-01A-11R-A22L-07	2.313397	Tumor
TCGA-DD-A1EB-01A-11R-A131-07	1.691751	Tumor
TCGA-BC-A10Z-01A-11R-A131-07	2.91762	Tumor
TCGA-BC-A216-01A-11R-A155-07	5.183955	Tumor
TCGA-BC-A10Y-01A-11R-A131-07	2.707116	Tumor
TCGA-BC-A10Q-01A-11R-A131-07	4.092977	Tumor
TCGA-FV-A3I1-01A-11R-A22L-07	2.950059	Tumor
TCGA-DD-A1EC-01A-21R-A131-07	3.030895	Tumor
TCGA-ES-A2HT-01A-12R-A180-07	0.79721	Tumor
TCGA-FV-A2QR-01A-11R-A213-07	2.022577	Tumor
TCGA-DD-A118-01A-11R-A131-07	1.952282	Tumor

Supplementary Table 3. Clinical data related to TCGA-LIHC

	Group I [Number]	Group J [Number]	Statistic t	Degrees of Freedom (df)	Difference (J-I)	95%CI	p
**status**	**Normal [50]**	**Tumor [374]**	**16.793**	**108.0609**	**0.816**	**0.719 - 0.912**	**0**
**T stage**	**T1 [183]**	**T2&T3&T4 [188]**	**14482**		**0.16**	**0.043 - 0.277**	**0.008**
**Pathologic.stage**	**Stage I&Stage II [260]**	**Stage III&Stage IV [90]**	**2.257**	**348**	**0.151**	**0.019 - 0.282**	**0.025**
**Histologic grade**	**G1 55**	**G2 178**	**2.471046**				**0.04**

	**G1**	**G3&G4 136**	**5.288321**				**0**
	**G2**	**G3&G4**	**4.072701**				**0**
**Tumor status**	**Tumor free [202]**	**With tumor [153]**	**2.621**	**353**	**0.152**	**0.038 - 0.266**	**0.009**
**age**	**<=60 [177]**	**>60 [196]**	**19778**		**-0.142**	**-0.238**	**0.019**
**weight**	**<=70 [184]**	**>70 [162]**	**-2.459**	**344**	**-0.143**	**-0.229**	**0.014**
**AFP**	**<=400 [215]**	**>400 [65]**	**3.328**	**278**	**0.246**	**0.101 - 0.392**	**0.001**

Supplementary Table 4. Information on the 377 clinical samples of HCC

Id	futime	fustat	age	gender	grade	stage	T	M	N
TCGA-DD-A1EA	2415	0	68	MALE	G2	Stage II	T2	M0	N0
TCGA-KR-A7K0	65	1	65	MALE	G1	Stage I	T1	M0	N0
TCGA-DD-A4NS	2456	1	61	FEMALE	G2	Stage I	T1	M0	N0
TCGA-CC-A5UC	347	1	63	MALE	G3	Stage IIIA	T3	M0	N0
TCGA-G3-AAV7	361	0	38	MALE	G2	Stage II	T2	M0	N0
TCGA-DD-AAED	763	0	51	MALE	G3	Stage I	T1	M0	N0
TCGA-DD-AAE0	555	0	45	FEMALE	G4	Stage IIIA	T3a	M0	N0
TCGA-CC-A3M9	300	1	45	MALE	G3	Stage IIIA	T3	M0	N0
TCGA-DD-A3A4	612	1	37	MALE	G3	Stage IIIA	T3	M0	N0
TCGA-DD-AADB	1242	0	51	MALE	G4	Stage I	T1	M0	N0
TCGA-G3-A25V	860	0	68	MALE	G2	Stage I	T1	M0	N0
TCGA-DD-AAE7	644	0	72	MALE	G2	Stage I	T1	M0	N0
TCGA-LG-A9QC	425	0	48	MALE	G2	Stage I	T1	M0	NX
TCGA-DD-AAEH	784	0	73	MALE	G2	Stage I	T1	M0	N0
TCGA-DD-AAW1	1989	0	55	MALE	G2	Stage IIIA	T3	M0	N0
TCGA-2Y-A9H9	697	0	70	MALE	G2	Stage I	T1	MX	N0
TCGA-5C-AAPD	20	0	61	MALE	G1	Stage II	T2	M0	N0
TCGA-EP-A2KA	357	0	52	FEMALE	G3	Stage IIIA	T3a	MX	NX
TCGA-DD-A73B	283	1	72	FEMALE	G2	Stage I	T1	M0	N0
TCGA-UB-A7MD	52	1	67	MALE	G3	Stage I	T1	MX	N0
TCGA-DD-A113	2425	0	55	FEMALE	G3	Stage II	T2	M0	N0
TCGA-5C-A9VH	322	0	70	MALE	G2	Stage I	T1	M0	N0
TCGA-ED-A5KG	482	0	60	FEMALE	G2	Stage II	T2	M0	N0
TCGA-BC-A10U	837	1	69	MALE	G2	unknow	T2	MX	NX
TCGA-DD-A39V	643	1	77	MALE	G3	Stage II	T2	M0	NX
TCGA-ED-A627	423	0	74	MALE	G2	Stage I	T1	M0	NX
TCGA-G3-AAV2	372	0	50	MALE	G1	Stage I	T1	M0	N0
TCGA-ZS-A9CD	1386	1	73	MALE	G2	Stage II	T2	MX	NX
TCGA-GJ-A9DB	67	1	68	MALE	G2	Stage I	T1	MX	N0
TCGA-G3-AAV4	27	1	83	FEMALE	G1	Stage I	T1	M0	N0
TCGA-DD-AADP	458	0	45	MALE	G3	Stage I	T1	M0	N0
TCGA-DD-A4NL	1711	0	46	MALE	G1	Stage I	T1	M0	N0
TCGA-CC-5261	97	1	44	MALE	G2	Stage II	T2	M0	N0
TCGA-UB-A7MC	500	0	59	MALE	G3	Stage IIIA	T3a	MX	N0
TCGA-CC-A7IF	649	1	59	MALE	G1	Stage IIIA	T3	M0	N0
TCGA-4R-AA8I	262	1	66	MALE	G2	Stage II	T2	MX	NX
TCGA-DD-A73C	701	0	65	FEMALE	G1	Stage IIIA	T3a	M0	N0
TCGA-CC-5259	250	0	60	FEMALE	G2	Stage IIIC	T4	M0	N0
TCGA-2Y-A9GW	1271	1	64	MALE	G2	Stage I	T1	MX	N0
TCGA-BC-A10S	1423	1	81	MALE	G1	unknow	T3	MX	NX
TCGA-UB-A7ME	486	0	51	MALE	G2	Stage I	T1	MX	NX
TCGA-ED-A97K	6	0	54	MALE	G2	Stage IIIA	T3a	M0	N0
TCGA-XR-A8TF	693	1	74	MALE	G1	Stage I	T1	MX	NX
TCGA-CC-A7II	399	0	54	MALE	G3	Stage IIIA	T3	M0	N0
TCGA-DD-AADQ	436	0	59	MALE	G3	Stage II	T2	M0	N0
TCGA-K7-A6G5	512	0	66	MALE	G2	Stage I	T1	MX	N0
TCGA-CC-5264	102	1	71	MALE	G2	Stage IIIA	T3	M0	N0
TCGA-DD-A39Y	171	1	67	MALE	G3	Stage I	T1	M0	NX
TCGA-ED-A8O6	56	1	50	FEMALE	G3	Stage IIIA	T3a	M0	N0
TCGA-DD-A1E9	2759	1	70	MALE	G2	Stage I	T1	M0	N0

TCGA-DD-A1EC	602	0	20	FEMALE	G3	Stage I	T1	M0	N0
TCGA-DD-A115	2542	1	53	MALE	G2	Stage IIIA	T3	M0	N0
TCGA-HP-A5MZ	91	1	78	MALE	G2	Stage I	T1	M0	NX
TCGA-DD-A1EK	558	1	64	FEMALE	G2	Stage IVB	T4	M1	N0
TCGA-DD-AAE2	638	0	51	MALE	G3	Stage I	T1	M0	N0
TCGA-CC-A5UE	272	1	48	MALE	G2	Stage IIIB	T4	M0	N0
TCGA-NI-A8LF	606	0	74	MALE	G3	Stage I	T1	MX	NX
TCGA-BW-A5NO	20	0	50	MALE	G2	Stage IIIA	T3a	MX	NX
TCGA-CC-A5UD	304	1	45	MALE	G2	Stage IIIA	T3	M0	N0
TCGA-DD-A1EF	394	1	57	FEMALE	G3	Stage I	T1	M0	N0
TCGA-GJ-A3OU	879	0	59	MALE	G2	Stage I	T1	MX	NX
TCGA-DD-AAVU	2202	0	46	MALE	G2	Stage II	T2	M0	N0
TCGA-G3-A6UC	671	0	65	MALE	G2	Stage IIIB	T3b	M0	N0
TCGA-CC-A9FU	0	0	52	FEMALE	G2	Stage IIIA	T3a	M0	N0
TCGA-DD-AAD3	1295	0	43	MALE	G2	Stage I	T1	M0	N0
TCGA-2Y-A9GU	1939	0	55	FEMALE	G2	Stage I	T1	MX	NX
TCGA-MI-A75G	698	0	63	MALE	G2	Stage II	T2	M0	N0
TCGA-FV-A4ZP	2486	1	78	MALE	G2	Stage IIIA	T3	M0	NX
TCGA-PD-A5DF	639	1	58	FEMALE	G2	Stage IIIB	T4	M0	N0
TCGA-DD-A3A9	931	1	64	FEMALE	G2	Stage IVB	T4	M1	N0
TCGA-DD-AACA	2301	0	65	MALE	G3	Stage I	T1	M0	N0
TCGA-DD-AAW2	1855	0	69	MALE	G2	Stage I	T1	M0	N0
TCGA-DD-AADD	1231	0	51	MALE	G4	Stage I	T1	M0	N0
TCGA-XR-A8TG	898	0	58	MALE	G2	Stage I	T1	M0	NX
TCGA-UB-AA0V	314	0	69	FEMALE	G1	Stage I	unknow	MX	NX
TCGA-CC-A7IE	217	1	57	MALE	G2	Stage IIIA	T3	M0	N0
TCGA-BC-A69I	387	0	69	MALE	G1	Stage I	T1	M0	N0
TCGA-DD-AAE3	566	0	50	MALE	G2	Stage I	T1	M0	N0
TCGA-CC-A1HT	101	1	50	MALE	G3	Stage IIIA	T3	M0	N0
TCGA-ZP-A9D1	21	0	56	FEMALE	G2	unknow	T1	MX	NX
TCGA-EP-A3JL	303	0	76	MALE	G2	Stage I	T1	MX	NX
TCGA-CC-A7IL	278	1	61	MALE	G1	Stage IIIA	T3	M0	N0
TCGA-CC-5263	129	1	35	MALE	G1	Stage IIIA	T3	M0	N0
TCGA-G3-A25X	1779	0	73	MALE	G3	Stage II	T2	M0	N0
TCGA-WQ-A9G7	30	0	71	FEMALE	G3	unknow	T3a	M0	NX
TCGA-G3-A25Z	655	0	58	MALE	G2	Stage I	T1	M0	N0
TCGA-DD-AACX	170	0	66	MALE	G3	Stage II	T2	M0	N0
TCGA-DD-AAE6	141	0	59	FEMALE	G2	Stage I	T1	M0	N0
TCGA-UB-AA0U	327	0	60	MALE	G2	Stage II	T2	MX	NX
TCGA-RG-A7D4	1098	0	69	MALE	G2	Stage II	T2	M0	N0
TCGA-MI-A75I	630	0	61	MALE	G1	unknow	T2	MX	NX
TCGA-DD-AAVX	1718	0	38	MALE	G2	Stage II	T2	M0	N0
TCGA-EP-A2KC	19	1	62	MALE	G3	Stage I	T1	MX	NX
TCGA-DD-AADN	898	0	59	MALE	G4	Stage I	T1	MX	NX
TCGA-DD-A4NV	2018	0	61	MALE	G1	Stage IIIA	T3	M0	N0
TCGA-T1-A6J8	23	0	68	MALE	G2	unknow	T1	M0	NX
TCGA-DD-A4NK	1210	1	80	FEMALE	G2	Stage IIIA	T3	M0	N0
TCGA-CC-A7IJ	382	0	56	MALE	G3	Stage II	T2	M0	N0
TCGA-DD-AADR	2028	0	58	MALE	G3	Stage I	T1	M0	N0
TCGA-DD-A4NH	690	0	65	FEMALE	G3	Stage IIIB	T3b	M0	N0
TCGA-BD-A2L6	1363	0	69	MALE	G2	unknow	T2	MX	NX
TCGA-G3-A3CG	673	0	80	MALE	G2	Stage I	T1	M0	N0
TCGA-RC-A6M3	0	0	24	MALE	G3	Stage II	T2	M0	N0
TCGA-DD-AACB	2324	0	74	FEMALE	G3	Stage I	T1	M0	N0
TCGA-BC-A10Y	711	1	76	MALE	G3	unknow	T4	MX	NX
TCGA-CC-A9FW	248	0	68	MALE	G2	Stage IIIA	T3	M0	N0
TCGA-DD-A1EL	415	1	23	MALE	G3	Stage II	T2	M0	N0
TCGA-DD-AAEE	810	0	55	MALE	G4	Stage I	T1	M0	N0
TCGA-BC-A10W	91	1	50	MALE	G3	unknow	T4	MX	NX
TCGA-CC-A8HU	344	1	39	FEMALE	G3	Stage IIIA	T3	M0	N0
TCGA-RC-A7SK	472	0	59	MALE	G3	Stage I	T1	M0	N0
TCGA-CC-5258	129	1	48	MALE	G2	Stage II	T2	M0	N0
TCGA-UB-A7MF	214	1	56	MALE	G2	Stage IIIA	T3a	MX	NX
TCGA-DD-AACL	107	1	66	FEMALE	G3	Stage I	T1	M0	N0
TCGA-BC-A10T	837	1	76	MALE	G1	unknow	T4	MX	NX
TCGA-DD-AACE	2184	0	62	MALE	G3	Stage I	T1	M0	N0
TCGA-BC-A110	2116	1	51	FEMALE	G1	unknow	T1	MX	NX

TCGA-ED-A7PY	390	0	20	FEMALE	G3	Stage II	T2	M0	NX
TCGA-DD-A4NR	9	1	85	FEMALE	G3	Stage I	T1	M0	N0
TCGA-BC-A8YO	562	0	66	FEMALE	G3	Stage IIIC	T4	M0	N0
TCGA-DD-AADU	554	0	60	MALE	G3	Stage II	T2	M0	N0
TCGA-BC-A10Z	34	1	62	FEMALE	G2	Stage I	T1	MX	N0
TCGA-DD-AACK	9	0	70	MALE	G2	Stage I	T1	M0	N0
TCGA-DD-AAEK	1067	0	51	MALE	G3	Stage II	T2	M0	N0
TCGA-DD-AAW3	1633	0	69	MALE	G2	Stage I	T1	M0	N0
TCGA-2Y-A9GZ	848	1	82	FEMALE	G2	Stage II	T2	MX	NX
TCGA-DD-A73F	1085	0	77	FEMALE	G1	Stage I	T1	M0	N0
TCGA-DD-AACS	1804	0	39	MALE	G3	Stage I	T1	M0	N0
TCGA-ED-A66Y	296	1	51	FEMALE	G3	Stage IIIA	T3a	M0	N0
TCGA-G3-A3CJ	594	0	52	MALE	G2	Stage II	T2	M0	N0
TCGA-2Y-A9H2	1731	0	64	FEMALE	G3	Stage I	T1	MX	N0
TCGA-ED-A7PZ	6	0	61	MALE	G2	Stage II	T2	M0	NX
TCGA-DD-AACI	1618	0	69	MALE	G3	Stage II	T2	M0	N0
TCGA-DD-A116	1622	1	68	MALE	G3	Stage IIIA	T3	M0	N0
TCGA-DD-AAEG	719	0	59	FEMALE	G3	Stage I	T1	M0	N0
TCGA-DD-A114	1149	1	42	MALE	G3	Stage II	T2	M0	unknow
TCGA-ZS-A9CF	2412	0	64	MALE	G2	Stage II	T2	MX	NX
TCGA-RC-A6M6	9	0	75	MALE	G3	Stage II	T2	M0	NX
TCGA-5R-AA1C	364	0	57	MALE	G2	Stage II	T2	M0	N0
TCGA-CC-5262	103	1	67	MALE	G1	Stage IIIC	T4	M0	N0
TCGA-DD-AACO	1876	0	40	MALE	G3	Stage I	T1	M0	N0
TCGA-ZP-A9CY	782	0	66	FEMALE	G1	unknow	T1	MX	NX
TCGA-ZP-A9D4	395	0	64	FEMALE	G1	unknow	T1	MX	NX
TCGA-ZS-A9CE	1241	0	79	FEMALE	G1	Stage II	T2	MX	NX
TCGA-DD-AAVS	1823	0	56	MALE	G2	Stage I	T1	M0	N0
TCGA-XR-A8TC	1339	0	43	FEMALE	G2	Stage I	T1	MX	NX
TCGA-DD-A39W	827	1	29	FEMALE	G2	Stage III	T3	M0	N0
TCGA-BC-A69H	444	0	64	MALE	G3	Stage II	T2	M0	NX
TCGA-DD-A4NA	1008	0	67	FEMALE	G3	Stage IIIC	T2	M0	N1
TCGA-MI-A75E	507	0	61	MALE	G2	Stage IIIC	T4	M0	N0
TCGA-DD-AAVZ	1900	0	38	MALE	G2	Stage I	T1	M0	N0
TCGA-G3-A5SL	621	0	70	MALE	G2	Stage II	T2	M0	NX
TCGA-ED-A7XP	400	0	53	FEMALE	G3	Stage II	T2	M0	N0
TCGA-EP-A2KB	334	0	46	FEMALE	G2	Stage I	T1	MX	NX
TCGA-DD-A4NI	561	0	67	MALE	G2	Stage II	T2	M0	NX
TCGA-CC-A9FV	0	0	57	MALE	G2	Stage IIIA	T3	M0	N0
TCGA-WQ-AB4B	395	0	62	MALE	G2	Stage II	T2	M0	NX
TCGA-G3-AAV1	359	1	51	MALE	G3	Stage IIIC	T4	M0	N0
TCGA-CC-A3MC	363	0	54	MALE	G2	Stage IIIA	T3	M0	N0
TCGA-G3-AAUZ	480	0	48	MALE	G2	Stage I	T1	M0	N0
TCGA-DD-AACM	1769	0	48	MALE	G3	Stage II	T2	M0	N0
TCGA-DD-A11D	1560	1	57	FEMALE	G2	Stage I	T1	M0	N0
TCGA-DD-AACF	365	1	68	MALE	G3	Stage I	T1	M0	N0
TCGA-BD-A3EP	409	0	75	FEMALE	G2	Stage I	T1	M0	N0
TCGA-ED-A4XI	386	0	58	MALE	G3	Stage II	T2	M0	N0
TCGA-DD-A11A	79	0	67	MALE	G3	Stage I	T1	M0	N0
TCGA-QA-A7B7	94	0	48	MALE	G2	Stage II	T2	MX	NX
TCGA-DD-AADL	636	0	58	MALE	G4	Stage I	T1	M0	N0
TCGA-2Y-A9H6	357	0	68	FEMALE	G2	Stage I	T1	MX	NX
TCGA-2Y-A9GY	757	1	64	FEMALE	G3	Stage II	T2	MX	NX
TCGA-DD-A73E	44	0	66	MALE	G1	Stage I	T1	M0	N0
TCGA-RC-A7SF	579	0	66	MALE	G2	Stage I	T1	M0	N0
TCGA-BC-A3KG	498	0	68	FEMALE	G3	Stage II	T2	M0	N0
TCGA-DD-AAVP	2752	0	48	MALE	G1	Stage I	T1	M0	N0
TCGA-ED-A459	408	0	47	MALE	G2	Stage II	T2	M0	N0
TCGA-DD-AACW	1424	0	43	MALE	G3	Stage I	T1	M0	N0
TCGA-3K-AAZ8	396	0	65	MALE	G1	Stage IIIB	T3b	MX	NX
TCGA-G3-A7M5	447	0	76	MALE	G2	Stage I	T1	MX	NX
TCGA-FV-A3R2	194	1	75	MALE	unknow	Stage I	T1	MX	NX
TCGA-BD-A3ER	1115	0	62	MALE	G2	Stage II	T2	MX	NX
TCGA-DD-AADY	555	0	55	FEMALE	G2	Stage I	T1	M0	N0
TCGA-DD-AACY	1450	0	61	MALE	G3	Stage I	T1	M0	N0
TCGA-DD-A1EI	183	0	46	MALE	G2	Stage I	T1	M0	N0
TCGA-2Y-A9HB	260	0	66	MALE	G2	Stage I	T1	MX	NX

TCGA-CC-A123	219	0	24	FEMALE	G1	Stage IIIA	T3	M0	N0
TCGA-XR-A8TE	925	0	16	MALE	G1	Stage IIIA	T3	MX	N0
TCGA-CC-A8HT	140	1	74	MALE	G2	Stage IIIA	T3	M0	N0
TCGA-G3-A5SM	520	0	58	MALE	G3	Stage II	T2	M0	NX
TCGA-ES-A2HS	688	1	80	MALE	G2	Stage I	T1	MX	NX
TCGA-FV-A2QQ	729	0	80	MALE	G2	Stage I	T1	MX	N0
TCGA-DD-AACU	1567	0	59	MALE	G3	Stage I	T1	M0	N0
TCGA-RC-A7SH	468	0	42	MALE	G3	Stage II	T2	M0	N0
TCGA-ZP-A9D2	743	0	51	MALE	G2	unknow	T2	MX	NX
TCGA-DD-AACT	1562	0	69	FEMALE	G2	Stage I	T1	M0	N0
TCGA-DD-AADG	1145	0	70	MALE	G3	Stage IIIA	T3a	M0	N0
TCGA-2Y-A9H0	3675	0	49	MALE	G1	Stage IIIA	T3	M0	N0
TCGA-G3-AAV6	65	1	53	FEMALE	G3	Stage IIIA	T3a	M0	N0
TCGA-DD-A73D	693	0	68	FEMALE	G1	Stage II	T2	MX	NX
TCGA-FV-A2QR	581	1	75	MALE	G1	Stage I	T1	M0	N0
TCGA-DD-AADK	1049	0	68	FEMALE	G3	Stage II	T2	M0	N0
TCGA-DD-A4NE	660	1	75	FEMALE	G3	Stage IIIA	T3a	M0	N0
TCGA-CC-A7IG	299	1	47	MALE	G2	Stage II	T2	M0	N0
TCGA-DD-A1EB	2017	0	72	FEMALE	G2	Stage I	T1	M0	N0
TCGA-DD-A118	3437	0	77	FEMALE	G2	Stage II	T2	M0	N0
TCGA-5R-AAAM	46	1	65	FEMALE	G2	Stage II	T2	M0	N0
TCGA-DD-AAVQ	2728	0	38	MALE	G2	Stage I	T1	M0	N0
TCGA-G3-A25U	1636	0	63	FEMALE	G3	Stage I	T1	M0	N0
TCGA-G3-A7M8	430	0	31	MALE	G1	Stage I	T1	MX	NX
TCGA-DD-A3A5	3125	1	66	FEMALE	G2	Stage III	T3	M0	N0
TCGA-DD-AACZ	171	1	63	FEMALE	G4	Stage I	T1	M0	N0
TCGA-BC-A217	421	0	75	FEMALE	G3	Stage II	T2	M0	NX
TCGA-ED-A7XO	427	0	29	MALE	G2	Stage IIIA	T3a	M0	N0
TCGA-FV-A495	1	0	51	FEMALE	G2	Stage II	T2	M0	NX
TCGA-DD-A1EH	1495	0	23	MALE	G3	Stage III	T3	M0	N0
TCGA-FV-A23B	1852	1	70	FEMALE	unknow	Stage II	T2	M0	N0
TCGA-MI-A75C	291	0	64	MALE	G3	Stage I	T1	M0	N0
TCGA-DD-A4NP	3104	0	32	MALE	G3	Stage I	T1	M0	N0
TCGA-DD-AADF	115	1	64	FEMALE	G4	Stage I	T1	M0	N0
TCGA-DD-AAEA	575	0	65	MALE	G3	Stage I	T1	M0	N0
TCGA-EP-A3RK	363	0	73	MALE	G2	Stage IIIA	T3a	MX	NX
TCGA-BC-4072	1490	1	74	FEMALE	G3	Stage IIIA	T3	M0	N0
TCGA-FV-A3I0	848	0	76	FEMALE	G2	Stage II	T2	M0	NX
TCGA-UB-A7MB	601	0	24	MALE	G3	Stage II	T2	MX	NX
TCGA-ZP-A9CZ	706	0	72	MALE	G1	unknow	T1	MX	NX
TCGA-MI-A75H	747	0	77	MALE	unknow	unknow	unknow	MX	NX
TCGA-G3-A5SJ	698	0	59	MALE	G2	Stage I	T1	M0	NX
TCGA-DD-AAD0	137	0	73	FEMALE	G2	Stage I	T1	M0	N0
TCGA-ED-A82E	408	0	60	FEMALE	G2	Stage IIIA	T3a	M0	N0
TCGA-BC-A5W4	547	1	69	MALE	G3	Stage IIIA	T3a	M0	NX
TCGA-DD-AAEI	1531	0	72	MALE	G2	Stage I	T1	M0	N0
TCGA-2Y-A9GT	1624	1	51	MALE	G2	Stage I	T1	MX	NX
TCGA-XR-A8TD	1030	0	49	FEMALE	G3	Stage IIIB	T3	M0	N0
TCGA-2Y-A9H4	1452	0	68	MALE	G2	Stage I	T1	MX	N0
TCGA-DD-AACD	381	1	48	MALE	G4	Stage I	T1	M0	N0
TCGA-BC-A112	153	1	80	MALE	G2	unknow	T3	MX	NX
TCGA-2Y-A9GV	2532	1	54	FEMALE	G1	Stage I	T1	MX	NX
TCGA-DD-AADJ	1066	0	70	FEMALE	G3	Stage I	T1	M0	N0
TCGA-CC-5260	87	1	61	FEMALE	G1	Stage IIIC	T4	M0	N0
TCGA-LG-A6GG	387	0	79	FEMALE	G2	Stage II	T2	M0	NX
TCGA-G3-AAV0	476	0	58	MALE	G2	Stage I	T1	M0	N0
TCGA-DD-AACQ	432	1	50	MALE	G3	Stage II	T2	M0	N0
TCGA-KR-A7K7	407	0	61	FEMALE	G1	Stage II	T2	M0	N0
TCGA-DD-A4NJ	760	0	54	FEMALE	G2	Stage II	T2	M0	N0
TCGA-DD-AAVV	2455	0	56	MALE	G3	Stage II	T2	M0	N0
TCGA-DD-AAC8	16	1	72	MALE	G3	Stage I	T1	M0	N0
TCGA-G3-A25W	935	0	79	FEMALE	G2	Stage IIIB	T3b	M0	N0
TCGA-WJ-A86L	345	0	68	FEMALE	G2	Stage I	T1	MX	NX
TCGA-CC-A3MB	315	1	36	MALE	G1	Stage IIIA	T3	M0	N0
TCGA-G3-A7M7	361	0	65	MALE	G1	Stage I	T1	MX	NX
TCGA-DD-AADC	425	1	53	MALE	G3	Stage I	T1	M0	N0
TCGA-DD-AACC	1685	1	61	MALE	G2	Stage I	T1	M0	N0

TCGA-G3-A25T	1553	0	45	FEMALE	G2	Stage IIIA	T3	M0	N0
TCGA-G3-A3CH	780	0	53	MALE	G2	Stage IIIA	T3a	M0	N0
TCGA-HP-A5N0	752	1	90	FEMALE	unknow	unknow	TX	M0	NX
TCGA-DD-A3A7	419	1	67	MALE	G3	Stage IIIB	T3b	M0	N0
TCGA-BC-4073	352	0	73	MALE	G3	Stage IIIA	T3	MX	N0
TCGA-2Y-A9HA	36	1	70	MALE	G2	Stage II	T2	MX	NX
TCGA-WX-AA44	615	0	64	FEMALE	G3	Stage I	T1	MX	NX
TCGA-DD-A39Z	601	1	43	FEMALE	G2	Stage II	T2	M0	NX
TCGA-DD-AAD5	1345	0	54	MALE	G3	Stage I	T1	M0	N0
TCGA-2Y-A9H7	1168	0	81	FEMALE	G2	Stage I	T1	MX	N0
TCGA-DD-A1EJ	1005	1	71	FEMALE	G2	Stage IIIC	T1	M0	N1
TCGA-DD-A4NG	802	1	77	MALE	G2	Stage IIIA	T3a	M0	NX
TCGA-CC-A9FS	211	0	55	MALE	G2	Stage II	T2	M0	N0
TCGA-DD-A4NO	2245	0	65	MALE	G1	Stage I	T1	M0	N0
TCGA-DD-AAVY	1970	0	56	MALE	G2	Stage IIIA	T3	M0	N0
TCGA-G3-A25Y	452	1	52	FEMALE	G3	Stage I	T1	M0	N0
TCGA-BC-A3KF	8	0	66	FEMALE	G2	Stage I	T1	M0	NX
TCGA-DD-A119	223	1	40	MALE	G3	Stage IV	T3a	M1	N0
TCGA-DD-AAVR	2513	0	44	MALE	G2	Stage I	T1	M0	N0
TCGA-UB-A7MA	535	0	62	FEMALE	G2	Stage II	T2b	M0	N0
TCGA-ZS-A9CG	341	0	55	MALE	G2	Stage II	T2	MX	NX
TCGA-ES-A2HT	438	1	54	MALE	G2	Stage I	T1	MX	NX
TCGA-G3-A7M9	56	1	70	MALE	G2	Stage IIIB	T3b	MX	NX
TCGA-DD-AADI	1085	0	43	FEMALE	G3	Stage I	T1	M0	N0
TCGA-CC-A7IK	262	1	59	MALE	G3	Stage IIIA	T3	M0	N0
TCGA-DD-AADW	587	0	48	MALE	G3	Stage I	T1	M0	N0
TCGA-FV-A4ZQ	12	0	52	MALE	G2	Stage I	T1	M0	NX
TCGA-DD-AADM	12	1	58	MALE	G3	Stage II	T2	M0	N0
TCGA-DD-AAW0	2015	0	54	MALE	G2	Stage I	T1	M0	N0
TCGA-K7-AAU7	359	0	61	MALE	G2	Stage II	T2a	MX	NX
TCGA-ED-A8O5	406	0	59	FEMALE	G3	Stage IIIA	T3a	M0	N0
TCGA-DD-A73G	3478	0	73	FEMALE	G3	Stage I	T1	M0	N0
TCGA-DD-A11B	14	1	73	MALE	G2	Stage I	T1	M0	N0
TCGA-WX-AA46	756	0	61	MALE	G1	Stage II	T2	MX	NX
TCGA-ED-A66X	406	0	35	MALE	G3	Stage IIIA	T3a	M0	N0
TCGA-BC-A10X	770	1	52	FEMALE	G2	Stage IIIA	T3a	MX	N0
TCGA-DD-AADS	474	0	63	MALE	G2	Stage I	T1	M0	N0
TCGA-MR-A520	229	0	58	MALE	G1	Stage I	T1	MX	NX
TCGA-LG-A9QD	366	0	68	MALE	G2	Stage IIIA	T3a	M0	N0
TCGA-FV-A496	10	0	84	FEMALE	G2	Stage I	T1	M0	NX
TCGA-5C-A9VG	328	0	58	MALE	G2	Stage II	T2	M0	N0
TCGA-BC-A10R	308	1	66	FEMALE	G2	unknow	T3	MX	NX
TCGA-DD-AA3A	410	1	81	FEMALE	G4	Stage I	T1	MX	N0
TCGA-DD-A73A	728	0	71	MALE	G2	Stage I	T1	M0	N0
TCGA-BW-A5NQ	0	0	63	MALE	G3	Stage I	T1	MX	NX
TCGA-DD-A4NB	391	0	25	MALE	G2	Stage I	T1	M0	N0
TCGA-DD-AADO	453	0	55	MALE	G3	Stage I	T1	M0	N0
TCGA-FV-A3R3	366	1	38	FEMALE	G2	Stage I	T1	MX	NX
TCGA-RC-A7SB	588	0	53	MALE	G2	Stage II	T2	M0	N0
TCGA-DD-AAD2	658	0	66	MALE	G2	Stage I	T1	M0	N0
TCGA-DD-A1EG	1372	1	76	MALE	G3	Stage I	T1	M0	N0
TCGA-G3-A3CI	180	0	71	MALE	G2	Stage I	T1	M0	N0
TCGA-DD-AAD1	564	0	51	FEMALE	G4	Stage I	T1	M0	N0
TCGA-DD-A11C	662	0	69	MALE	G3	Stage I	T1	M0	N0
TCGA-WX-AA47	556	1	33	FEMALE	G2	Stage IIIA	T3a	MX	NX
TCGA-NI-A4U2	1791	1	71	MALE	G1	Stage IIIA	T3	MX	NX
TCGA-2Y-A9H3	1516	0	45	MALE	G1	Stage II	T2	MX	NX
TCGA-BC-A216	1351	0	62	FEMALE	G2	Stage IIIA	T3	M0	NX
TCGA-2Y-A9H5	555	1	59	FEMALE	G3	Stage I	T1	MX	N0
TCGA-EP-A12J	330	0	62	MALE	G1	Stage I	T1	MX	NX
TCGA-DD-AACP	415	0	64	MALE	G3	Stage I	T1	M0	N0
TCGA-DD-A3A2	2131	1	76	FEMALE	G1	Stage I	T1	M0	N0
TCGA-DD-A3A0	785	1	70	MALE	G2	Stage I	T1	M0	NX
TCGA-DD-A4ND	2232	0	56	FEMALE	G3	Stage I	T1	M0	N0
TCGA-G3-AAV5	354	0	67	MALE	G2	Stage II	T2	M0	N0
TCGA-DD-AAEB	478	0	60	MALE	G2	Stage I	T1	M0	N0
TCGA-2Y-A9H8	633	1	85	FEMALE	G2	unknow	T1	MX	NX

TCGA-G3-A25S	416	1	64	MALE	G2	Stage I	T1	M0	N0
TCGA-RC-A6M5	15	0	20	FEMALE	G2	Stage IVA	T1	M0	N1
TCGA-DD-AAE4	608	0	49	FEMALE	G1	Stage I	T1	M0	N0
TCGA-DD-AACH	195	1	69	MALE	G3	Stage II	T2	M0	N0
TCGA-DD-AAD8	1219	0	73	FEMALE	G2	Stage I	T1	M0	N0
TCGA-CC-A7IH	365	0	58	MALE	G1	Stage IIIA	T3	M0	N0
TCGA-DD-AAE9	722	0	69	MALE	G3	Stage I	T1	M0	N0
TCGA-DD-AADA	1233	0	66	FEMALE	G3	Stage I	T1	M0	N0
TCGA-DD-A39X	1694	1	78	FEMALE	G2	Stage I	T1	M0	NX
TCGA-DD-AADE	1202	0	50	MALE	G4	Stage I	T1	M0	N0
TCGA-K7-A5RG	519	0	66	MALE	G1	Stage I	T1	MX	NX
TCGA-DD-AAVW	2317	0	35	MALE	G2	Stage I	T1	M0	N0
TCGA-G3-A5SI	768	1	44	MALE	G2	Stage II	T2	M0	N0
TCGA-MR-A8JO	330	0	34	MALE	G3	Stage I	T1	MX	N0
TCGA-DD-AAE1	552	0	52	MALE	G3	Stage I	T1	M0	N0
TCGA-DD-A4NN	899	1	56	FEMALE	G3	Stage I	T1	M0	N0
TCGA-BW-A5NP	0	0	26	FEMALE	G3	Stage IV	T2	M1	N0
TCGA-CC-A8HV	279	1	51	FEMALE	G2	Stage II	T2	M0	N0
TCGA-G3-A3CK	585	0	61	MALE	G2	Stage I	T1	M0	N0
TCGA-EP-A26S	237	0	70	MALE	G2	Stage I	T1	MX	N0
TCGA-2Y-A9GX	2442	0	68	MALE	G2	Stage I	T1	MX	NX
TCGA-RC-A7S9	640	0	47	FEMALE	G3	Stage I	T1	M0	N0
TCGA-YA-A8S7	412	1	68	MALE	G3	Stage IIIA	T3a	MX	N0
TCGA-DD-A3A3	535	1	45	MALE	G2	Stage I	T1	M0	N0
TCGA-O8-A75V	538	0	54	MALE	G2	Stage I	T1	MX	NX
TCGA-ZP-A9D0	717	0	67	FEMALE	G1	unknow	T1	MX	NX
TCGA-DD-A1EE	349	1	73	MALE	G3	Stage IIIA	T3	M0	N0
TCGA-DD-A3A6	3258	1	72	FEMALE	G2	Stage II	T2	M0	N0
TCGA-RC-A6M4	22	0	74	FEMALE	G2	Stage IIIA	T3	MX	NX
TCGA-DD-AAD6	672	0	66	MALE	G3	Stage IIIA	T3a	M0	N0
TCGA-K7-A5RF	631	0	64	MALE	G1	Stage I	T1	MX	NX
TCGA-CC-A8HS	300	1	18	MALE	G1	Stage IIIC	T3	M0	N1
TCGA-G3-A5SK	744	0	58	MALE	G1	Stage I	T1	M0	NX
TCGA-DD-A4NQ	373	1	60	MALE	G3	Stage II	T2	M0	N0
TCGA-DD-A1ED	2301	0	68	MALE	G1	Stage I	T1	M0	N0
TCGA-G3-AAV3	412	0	58	FEMALE	G2	Stage II	T2	M0	N0
TCGA-ZP-A9CV	1088	1	59	MALE	G1	unknow	T1	MX	NX
TCGA-DD-AACV	1531	0	53	MALE	G3	Stage I	T1	M0	N0
TCGA-DD-AACG	469	1	52	MALE	G4	Stage II	T2	M0	N0
TCGA-CC-A3MA	303	1	61	MALE	G2	Stage IIIA	T3	M0	N0
TCGA-2V-A95S		0	unknow	MALE	G3	Stage II	T2	MX	NX
TCGA-2Y-A9H1	1229	1	58	MALE	G2	Stage I	T1	MX	NX
TCGA-DD-A4NF	428	0	72	MALE	G2	Stage I	T1	M0	N0
TCGA-DD-A3A1	233	1	65	MALE	G2	Stage IIIA	T3b	M0	N0
TCGA-KR-A7K2	657	0	64	MALE	G1	Stage I	T1	M0	N0
TCGA-DD-AAC9	347	0	51	MALE	G2	Stage I	T1	M0	N0
TCGA-DD-AACJ	2102	0	75	MALE	G2	Stage II	T2	M0	N0
TCGA-DD-AACN	1302	0	32	MALE	G3	Stage I	T1	M0	N0
TCGA-GJ-A6C0	31	1	75	FEMALE	G2	Stage II	T2	MX	NX
TCGA-DD-AAE8	664	0	45	MALE	G3	Stage I	T1	M0	N0
TCGA-DD-AADV	574	0	50	MALE	G3	Stage I	T1	M0	N0
TCGA-FV-A3I1	247	1	81	FEMALE	G2	Stage II	T2	MX	N0
TCGA-BC-A10Q	1135	1	72	FEMALE	unknow	unknow	T2	MX	NX
TCGA-2Y-A9GS	724	1	58	MALE	G2	unknow	T2	MX	NX
TCGA-ED-A7PX	6	0	48	FEMALE	G3	Stage II	T2	M0	NX
TCGA-DD-A3A8	11	1	75	MALE	G2	Stage II	T2	M0	N0
TCGA-G3-A7M6	632	0	60	FEMALE	G3	Stage I	T1	MX	NX
TCGA-KR-A7K8	906	0	57	MALE	G1	Stage I	T1	M0	N0
TCGA-5R-AA1D	337	0	17	FEMALE	G3	Stage IIIA	T3a	M0	N0

Supplementary Table 5. Univariate and multivariate analyses of prognostic factors（*TCERG1*）

	Univariate analysis			Multivariate analysis		
Parameter	HR	95% CI	pvalue	HR	95% CI	pvalue
age	1.01	0.98-1.02	0.591	1.01	0.99-1.03	0.260
gender	0.78	0.49-1.25	0.301	1.10	0.65-1.85	0.719
grade	1.02	0.75-1.39	0.914	1.02	0.72-1.44	0.914
stage	1.86	1.46-2.39	8.07E-07	1.19	0.43-3.24	0.738
T	1.80	1.43-2.27	4.73E-08	1.49	0.60-3.72	0.389
M	3.84	1.21-12.28	0.023	1.93	0.50-7.47	0.339
N	2.02	0.49-8.28	0.328	1.02	0.16-6.22	0.982
TCERG1	1.29	1.12-1.48	0.000	1.30	1.11-1.52	0.001

Supplementary Table 6. Genes co-expressed in the CCLE database and *TCERG1*

gene1	gene2	cor	pvalue
TCERG1	AGRN	-0.64	0.000571754
TCERG1	DNAJC11	0.652	0.000409761
TCERG1	TARDBP	0.757	1.20E-05
TCERG1	MRTO4	0.661	0.000321145
TCERG1	ZBTB40	0.682	0.000171955
TCERG1	SRSF10	0.706	8.13E-05
TCERG1	RP3-469D22.1	0.657	0.000356041
TCERG1	RCC1	0.654	0.000395815
TCERG1	SNRNP40	0.715	5.98E-05
TCERG1	ZMYM6NB	-0.72	4.92E-05
TCERG1	SFPQ	0.661	0.000318756
TCERG1	ZC3H12A	-0.627	0.000798223
TCERG1	INPP5B	0.681	0.000180677
TCERG1	HECTD3	-0.712	6.45E-05
TCERG1	MMACHC	0.621	0.000922092
TCERG1	ORC1	0.619	0.000978739
TCERG1	PRPF38A	0.701	9.45E-05
TCERG1	NDC1	0.645	0.000502213
TCERG1	JUN	-0.691	0.00013247
TCERG1	TM2D1	-0.659	0.000336366
TCERG1	JAK1	-0.63	0.000730225
TCERG1	LEPROT	-0.63	0.000743966
TCERG1	CDC7	0.687	0.000146554
TCERG1	SASS6	0.737	2.65E-05

TCERG1	CDC14A	0.633	0.00067536
TCERG1	OLFM3	0.627	0.000797045
TCERG1	ATXN7L2	0.643	0.000529529
TCERG1	CHI3L2	-0.637	0.000624136
TCERG1	WDR77	0.693	0.000124821
TCERG1	FAM72D	0.636	0.000629822
TCERG1	CHD1L	0.619	0.000971919
TCERG1	ILF2	0.645	0.000505014
TCERG1	PBXIP1	-0.656	0.000371486
TCERG1	TOMM40L	0.622	0.000897658
TCERG1	DUSP12	0.635	0.000653483
TCERG1	RP11-9L18.2	0.664	0.000294403
TCERG1	POU2F1	0.625	0.000833637
TCERG1	KIFAP3	-0.702	9.31E-05
TCERG1	LHX4	0.678	0.000195483
TCERG1	RP5-1180C10.2	0.65	0.000437672
TCERG1	DHX9	0.633	0.000686435
TCERG1	CSRP1	-0.694	0.000117684
TCERG1	SNRPE	0.633	0.000691638
TCERG1	SNRPGP10	0.761	1.01E-05
TCERG1	RRP15	0.721	4.76E-05
TCERG1	MARK1	0.638	0.000604208
TCERG1	LIN9	0.7	9.81E-05
TCERG1	PARP1	0.64	0.000567844
TCERG1	ZNF678	0.658	0.000348043
TCERG1	URB2	0.636	0.000638442
TCERG1	HEATR1	0.653	0.000403078
TCERG1	HNRNPU	0.713	6.23E-05
TCERG1	AHCTF1	0.694	0.00012021
TCERG1	LAPTM4A	-0.659	0.000343254
TCERG1	RBKS	-0.74	2.36E-05
TCERG1	CRIM1	-0.634	0.000661873
TCERG1	SRSF7	0.653	0.000399647
TCERG1	CHAC2	0.733	3.08E-05
TCERG1	FANCL	0.619	0.000976318
TCERG1	XPO1	0.645	0.000504628
TCERG1	PNO1	0.646	0.00048656
TCERG1	APLF	-0.627	0.000799076
TCERG1	SNRPG	0.808	1.05E-06
TCERG1	FAM136A	0.68	0.000181959
TCERG1	ALMS1-IT1	0.657	0.000364302
TCERG1	RP11-711M9.1	0.674	0.000222814
TCERG1	MAT2A	0.668	0.000262091
TCERG1	POLR1A	0.746	1.90E-05
TCERG1	RPIA	0.745	1.92E-05
TCERG1	NCAPH	0.655	0.000377497
TCERG1	MRPS9	0.681	0.000177299
TCERG1	LIMS1	-0.656	0.000372327
TCERG1	CCDC138	0.625	0.000839246
TCERG1	POLR1B	0.648	0.000462793

TCERG1	RP11-395L14.17	0.693	0.000124888
TCERG1	BBS5	-0.667	0.000273681
TCERG1	HOXD13	0.668	0.000265458
TCERG1	ZC3H15	0.762	9.47E-06
TCERG1	AC064834.1	0.64	0.000566423
TCERG1	NOP58	0.668	0.000266756
TCERG1	RQCD1	0.63	0.000737051
TCERG1	ZNF142	0.668	0.000260582
TCERG1	NCL	0.678	0.000195206
TCERG1	COPS7B	0.647	0.000474598
TCERG1	PASK	0.639	0.000583131
TCERG1	MRPS25	0.626	0.000822407
TCERG1	KIF15	0.791	2.51E-06
TCERG1	ELP6	0.623	0.000883693
TCERG1	DHX30	0.736	2.77E-05
TCERG1	QRICH1	0.625	0.000844425
TCERG1	VPRBP	0.682	0.000173065
TCERG1	RAD54L2	0.763	9.33E-06
TCERG1	MINA	0.644	0.000518564
TCERG1	TRMT10C	0.704	8.72E-05
TCERG1	C3orf17	0.674	0.000222366
TCERG1	KIAA2018	0.642	0.000545989
TCERG1	NAA50	0.632	0.000703211
TCERG1	QTRTD1	0.807	1.07E-06
TCERG1	POLQ	0.712	6.52E-05
TCERG1	IQCB1	0.69	0.000136839
TCERG1	CCDC58	0.666	0.000279314
TCERG1	MCM2	0.733	3.03E-05
TCERG1	ABTB1	-0.658	0.000349439
TCERG1	RUVBL1	0.643	0.000520706
TCERG1	CNBP	0.629	0.000756773
TCERG1	MRPL3	0.63	0.000733874
TCERG1	TOPBP1	0.659	0.00034328
TCERG1	DBR1	0.634	0.000665234
TCERG1	U2SURP	0.665	0.000289704
TCERG1	ARHGEF26	0.672	0.000233849
TCERG1	SETP14	0.631	0.000721166
TCERG1	PA2G4P4	0.696	0.000112658
TCERG1	USP13	0.656	0.000366181
TCERG1	TRA2B	0.831	2.67E-07
TCERG1	RFC4	0.628	0.000782936
TCERG1	LEPREL1	-0.684	0.000165598
TCERG1	PIGX	-0.623	0.000886975
TCERG1	ABCA11P	0.775	5.47E-06
TCERG1	ZNF721	0.695	0.000114423
TCERG1	RP11-440L14.1	0.71	6.98E-05
TCERG1	LETM1	0.626	0.000806451
TCERG1	NELFA	0.653	0.000403579
TCERG1	NOP14	0.706	8.00E-05
TCERG1	LYAR	0.713	6.35E-05

TCERG1	TBC1D14	0.662	0.000314267
TCERG1	TADA2B	0.621	0.000919424
TCERG1	FAM114A1	-0.633	0.000678381
TCERG1	GUF1	0.627	0.000804817
TCERG1	RCHY1	0.629	0.00075419
TCERG1	NUP54	0.636	0.000636801
TCERG1	RP11-33B1.1	0.647	0.000476718
TCERG1	PLK4	0.654	0.000387281
TCERG1	ABCE1	0.66	0.000334617
TCERG1	RP11-218F10.3	0.683	0.000167103
TCERG1	NUP155	0.628	0.000773574
TCERG1	PPWD1	0.619	0.000958756
TCERG1	GTF2H2C	0.814	7.36E-07
TCERG1	SMN1	0.63	0.000729224
TCERG1	BDP1	0.647	0.000474184
TCERG1	FAM174A	-0.668	0.000260351
TCERG1	STARD4	0.785	3.41E-06
TCERG1	LMNB1	0.823	4.29E-07
TCERG1	ISOC1	0.635	0.000651157
TCERG1	HSPA4	0.656	0.000373996
TCERG1	DDX46	0.776	5.07E-06
TCERG1	HNRNPA0	0.765	8.57E-06
TCERG1	CDC23	0.681	0.000177652
TCERG1	MATR3	0.686	0.000153204
TCERG1	CTC-329D1.2	0.645	0.000504142
TCERG1	ANKHD1	0.771	6.40E-06
TCERG1	PCDHGA10	0.62	0.000951227
TCERG1	KIAA0141	0.632	0.000699688
TCERG1	RBM27	0.714	6.09E-05
TCERG1	TCERG1	1	0
TCERG1	GRPEL2	0.679	0.000187321
TCERG1	PPARGC1B	0.686	0.000153024
TCERG1	TCOF1	0.721	4.80E-05
TCERG1	TNIP1	-0.645	0.000496937
TCERG1	LARP1	0.776	5.25E-06
TCERG1	GEMIN5	0.798	1.73E-06
TCERG1	SFXN1	0.702	9.10E-05
TCERG1	NOP16	0.76	1.04E-05
TCERG1	GPRIN1	0.669	0.000252107
TCERG1	DDX41	0.621	0.000931454
TCERG1	RMND5B	0.707	7.70E-05
TCERG1	AACSP1	0.664	0.000293846
TCERG1	HNRNPH1	0.703	8.77E-05
TCERG1	MAML1	0.759	1.10E-05
TCERG1	PRPF4B	0.645	0.000493577
TCERG1	RIOK1	0.712	6.61E-05
TCERG1	PAK1IP1	0.684	0.000160859
TCERG1	NEDD9	-0.624	0.000856245
TCERG1	NUP153	0.712	6.58E-05
TCERG1	PPP1R10	0.647	0.000468942

TCERG1	DDX39B	0.751	1.55E-05
TCERG1	BRD2	0.65	0.000431097
TCERG1	SNRPC	0.67	0.000249911
TCERG1	TAF11	0.636	0.000638563
TCERG1	FKBP5	0.624	0.000852728
TCERG1	SRSF3	0.824	4.04E-07
TCERG1	TAF8	0.725	4.20E-05
TCERG1	XPO5	0.774	5.74E-06
TCERG1	AARS2	0.637	0.000612218
TCERG1	MCM3	0.752	1.47E-05
TCERG1	MTO1	0.768	7.38E-06
TCERG1	PHIP	0.652	0.000415
TCERG1	SYNCRIP	0.619	0.000959518
TCERG1	MDN1	0.748	1.73E-05
TCERG1	NDUFAF4	0.643	0.000533733
TCERG1	MCM9	0.652	0.000416126
TCERG1	TRMT11	0.677	0.00020198
TCERG1	BCLAF1	0.656	0.000372859
TCERG1	ADAT2	0.67	0.000246922
TCERG1	MAFK	-0.618	0.000993935
TCERG1	KLHL7	-0.698	0.000103553
TCERG1	CCDC126	-0.624	0.000850196
TCERG1	TAX1BP1	-0.646	0.000489337
TCERG1	HERPUD2	-0.729	3.60E-05
TCERG1	STK17A	-0.677	0.000203725
TCERG1	ZNF107	0.724	4.34E-05
TCERG1	ZNF138	0.666	0.00027717
TCERG1	ZNF273	0.699	0.000101633
TCERG1	POM121	0.687	0.000150455
TCERG1	POM121C	0.631	0.000725551
TCERG1	DBF4	0.659	0.000341112
TCERG1	SRRT	0.728	3.73E-05
TCERG1	HBP1	-0.67	0.000245759
TCERG1	RBM28	0.656	0.000365344
TCERG1	EZH2	0.649	0.000449296
TCERG1	PAXIP1	0.65	0.000441648
TCERG1	NOM1	0.715	6.00E-05
TCERG1	ATP6V1H	-0.627	0.000801104
TCERG1	IMPAD1	-0.634	0.000663901
TCERG1	RBM12B	0.697	0.000106506
TCERG1	JRK	0.705	8.28E-05
TCERG1	C9orf41	0.643	0.000527094
TCERG1	HNRNPK	0.68	0.000182862
TCERG1	IARS	0.62	0.000947868
TCERG1	ANP32B	0.649	0.000445112
TCERG1	TEX10	0.628	0.000768792
TCERG1	SET	0.666	0.000279341
TCERG1	NUP188	0.664	0.000293853
TCERG1	FUBP3	0.629	0.000748977
TCERG1	EXOSC2	0.719	5.19E-05

TCERG1	ITGB1	-0.641	0.000554733
TCERG1	BMS1	0.707	7.87E-05
TCERG1	TFAM	0.736	2.76E-05
TCERG1	HNRNPH3	0.784	3.62E-06
TCERG1	DNA2	0.68	0.000182697
TCERG1	TET1	0.646	0.000480642
TCERG1	CCAR1	0.681	0.000178184
TCERG1	DDX21	0.659	0.00034145
TCERG1	SLC35G1	0.722	4.66E-05
TCERG1	HELLS	0.656	0.000372772
TCERG1	RP11-175O19.4	0.662	0.000312446
TCERG1	ARHGAP19	0.771	6.61E-06
TCERG1	CHUK	0.623	0.000889938
TCERG1	CWF19L1	0.631	0.000711782
TCERG1	SFXN3	-0.643	0.000521372
TCERG1	RP11-108L7.15	0.657	0.000362968
TCERG1	NPM3	0.666	0.000278972
TCERG1	PPRC1	0.76	1.05E-05
TCERG1	NOLC1	0.724	4.35E-05
TCERG1	TAF5	0.695	0.000115391
TCERG1	PDCD11	0.66	0.000334283
TCERG1	DCLRE1A	0.738	2.55E-05
TCERG1	NHLRC2	0.624	0.000853249
TCERG1	TIAL1	0.725	4.20E-05
TCERG1	SEC23IP	0.627	0.000792794
TCERG1	C10orf137	0.719	5.17E-05
TCERG1	BCCIP	0.7	9.74E-05
TCERG1	FANK1	-0.623	0.000880342
TCERG1	MTG1	0.723	4.53E-05
TCERG1	SPRN	0.669	0.000252435
TCERG1	IFITM3	-0.632	0.000698929
TCERG1	E2F8	0.644	0.000507005
TCERG1	PRMT3	0.718	5.32E-05
TCERG1	SVIP	0.619	0.000964224
TCERG1	NAT10	0.69	0.000136051
TCERG1	CELF1	0.676	0.000207665
TCERG1	NDUFS3	0.649	0.000447784
TCERG1	NUP160	0.728	3.70E-05
TCERG1	SSRP1	0.677	0.000201722
TCERG1	PRPF19	0.71	6.92E-05
TCERG1	CPSF7	0.669	0.000258589
TCERG1	FEN1	0.685	0.000156884
TCERG1	INCENP	0.755	1.31E-05
TCERG1	MTA2	0.673	0.000229449
TCERG1	SNHG1	0.683	0.000168071
TCERG1	TRMT112	0.672	0.000235851
TCERG1	SF1	0.781	4.00E-06
TCERG1	CDCA5	0.7	9.69E-05
TCERG1	RBM14	0.796	1.98E-06
TCERG1	SNORD15B	0.715	5.98E-05

TCERG1	TMEM126B	0.626	0.000814843
TCERG1	EED	0.66	0.000328993
TCERG1	KIAA1731	0.621	0.000925157
TCERG1	TIMM8B	0.646	0.000485242
TCERG1	ZNF202	0.655	0.000381438
TCERG1	RP11-677M14.6	0.621	0.000935205
TCERG1	PRDM10	0.758	1.12E-05
TCERG1	NCAPD3	0.742	2.15E-05
TCERG1	SCARNA12	0.628	0.000779658
TCERG1	KRAS	0.641	0.000554821
TCERG1	YAF2	0.632	0.000696153
TCERG1	ADAMTS20	0.634	0.000660627
TCERG1	PUS7L	0.672	0.000233635
TCERG1	SENP1	0.678	0.000194712
TCERG1	KANSL2	0.664	0.000292584
TCERG1	CCNT1	0.747	1.77E-05
TCERG1	DDX23	0.715	5.90E-05
TCERG1	KMT2D	0.754	1.37E-05
TCERG1	TROAP	0.621	0.000930391
TCERG1	MCRS1	0.708	7.42E-05
TCERG1	SMARCD1	0.635	0.000647957
TCERG1	ATF1	0.74	2.34E-05
TCERG1	PCBP2	0.632	0.000705891
TCERG1	TARBP2	0.685	0.000159103
TCERG1	HOXC13	0.637	0.000610292
TCERG1	RP11-968A15.2	0.651	0.000421836
TCERG1	RP11-968A15.8	0.638	0.000606208
TCERG1	HNRNPA1	0.669	0.000257728
TCERG1	PA2G4	0.743	2.07E-05
TCERG1	PRIM1	0.697	0.000106724
TCERG1	MBD6	0.685	0.000158483
TCERG1	NUP107	0.687	0.000149436
TCERG1	CPSF6	0.813	7.64E-07
TCERG1	CCDC59	0.759	1.09E-05
TCERG1	ALX1	0.658	0.000351216
TCERG1	UBE2N	0.699	0.000100661
TCERG1	MRPL42	0.704	8.58E-05
TCERG1	CCDC41	0.704	8.63E-05
TCERG1	METAP2	0.718	5.35E-05
TCERG1	NTN4	-0.645	0.000506061
TCERG1	SNRPF	0.732	3.25E-05
TCERG1	TMPO	0.704	8.47E-05
TCERG1	UTP20	0.765	8.35E-06
TCERG1	POLR3B	0.738	2.56E-05
TCERG1	SART3	0.822	4.66E-07
TCERG1	ANAPC7	0.787	3.05E-06
TCERG1	PPP1CC	0.674	0.000220193
TCERG1	ATXN2	0.637	0.000614654
TCERG1	PCNPP1	0.627	0.000786996
TCERG1	NAA25	0.748	1.70E-05

TCERG1	DDX54	0.636	0.000638316
TCERG1	RBM19	0.702	9.33E-05
TCERG1	RFC5	0.72	4.89E-05
TCERG1	RAB35	0.705	8.42E-05
TCERG1	GCN1L1	0.696	0.000111884
TCERG1	PXN-AS1	0.686	0.00015156
TCERG1	SRSF9	0.743	2.08E-05
TCERG1	C12orf43	0.654	0.00039632
TCERG1	CAMKK2	0.634	0.000672272
TCERG1	ANAPC5	0.642	0.000537532
TCERG1	KDM2B	0.688	0.000145276
TCERG1	DDX55	0.668	0.000263685
TCERG1	RAN	0.678	0.000198653
TCERG1	SFSWAP	0.716	5.76E-05
TCERG1	EP400NL	0.684	0.000163554
TCERG1	POLE	0.647	0.000471384
TCERG1	GTF3A	0.674	0.000222878
TCERG1	AL137059.1	0.637	0.000616513
TCERG1	BRCA2	0.714	6.04E-05
TCERG1	PDS5B	0.628	0.000775272
TCERG1	EXOSC8	0.769	7.09E-06
TCERG1	KBTBD6	0.62	0.000942874
TCERG1	NUFIP1	0.644	0.000508087
TCERG1	HNRNPA1L2	0.676	0.000207347
TCERG1	RBM26	0.676	0.000210959
TCERG1	HNRNPC	0.672	0.000231181
TCERG1	METTL3	0.68	0.000184575
TCERG1	PABPN1	0.72	4.94E-05
TCERG1	PNN	0.673	0.000227089
TCERG1	POLE2	0.667	0.000267152
TCERG1	SMEK1	0.709	7.17E-05
TCERG1	VRK1	0.696	0.000111664
TCERG1	EIF5	0.743	2.09E-05
TCERG1	XRCC3	0.775	5.49E-06
TCERG1	RP11-73M18.9	0.742	2.15E-05
TCERG1	HERC2P9	0.638	0.000607129
TCERG1	MEIS2	0.647	0.000469767
TCERG1	MGA	0.669	0.000256331
TCERG1	GABPB1	0.691	0.000132998
TCERG1	SMAD3	-0.644	0.000519614
TCERG1	HMG20A	0.661	0.000319694
TCERG1	FANCI	0.635	0.000657102
TCERG1	POLG	0.638	0.000595101
TCERG1	TICRR	0.706	8.04E-05
TCERG1	CRTC3	0.654	0.00039491
TCERG1	BLM	0.721	4.84E-05
TCERG1	CHD2	0.744	2.03E-05
TCERG1	SNRPA1	0.716	5.80E-05
TCERG1	AC005363.9	0.744	2.04E-05
TCERG1	RP11-304L19.11	0.619	0.00097188

TCERG1	C16orf59	0.681	0.000180108
TCERG1	SH2B1	0.649	0.000448781
TCERG1	RP11-146F11.1	0.718	5.32E-05
TCERG1	SRCAP	0.685	0.000160153
TCERG1	FUS	0.638	0.000605918
TCERG1	ORC6	0.625	0.000838121
TCERG1	SIAH1	0.632	0.000706787
TCERG1	HEATR3	0.785	3.43E-06
TCERG1	HNRNPA1P48	0.664	0.000292265
TCERG1	NUDT21	0.673	0.000225696
TCERG1	OGFOD1	0.634	0.000658289
TCERG1	NUP93	0.641	0.000551889
TCERG1	CIAPIN1	0.688	0.000145123
TCERG1	KIFC3	-0.682	0.000175599
TCERG1	CSNK2A2	0.648	0.000465234
TCERG1	CNOT1	0.692	0.000127562
TCERG1	NAE1	0.727	3.92E-05
TCERG1	CBFB	0.699	0.000101981
TCERG1	E2F4	0.768	7.25E-06
TCERG1	CTCF	0.797	1.84E-06
TCERG1	EDC4	0.754	1.33E-05
TCERG1	CTRL	0.752	1.44E-05
TCERG1	CIRH1A	0.658	0.000348339
TCERG1	NIP7	0.71	7.13E-05
TCERG1	TERF2	0.731	3.37E-05
TCERG1	DDX19A	0.654	0.000388696
TCERG1	SF3B3	0.747	1.76E-05
TCERG1	DHODH	0.65	0.000433749
TCERG1	TXNL4B	0.624	0.000849338
TCERG1	DHX38	0.706	8.01E-05
TCERG1	RFWD3	0.737	2.63E-05
TCERG1	KARS	0.74	2.38E-05
TCERG1	RPL18P13	0.643	0.000523445
TCERG1	CNTNAP4	0.691	0.000130421
TCERG1	MON1B	0.733	3.06E-05
TCERG1	RN7SL381P	0.671	0.000243227
TCERG1	KLHDC4	0.619	0.000968016
TCERG1	FLJ00104	0.623	0.000882056
TCERG1	RNF166	0.62	0.00093688
TCERG1	FANCA	0.744	1.98E-05
TCERG1	AFG3L1P	0.779	4.46E-06
TCERG1	PRPF8	0.646	0.000487715
TCERG1	METTL16	0.666	0.000278313
TCERG1	GSG2	0.628	0.000771678
TCERG1	ANKFY1	0.66	0.00032672
TCERG1	DHX33	0.653	0.000403223
TCERG1	POLR2A	0.627	0.000794255
TCERG1	PFAS	0.67	0.000244889
TCERG1	AC005822.1	0.631	0.000719585
TCERG1	ATAD5	0.661	0.000321603

TCERG1	ZNF207	0.636	0.000634738
TCERG1	GRN	-0.746	1.90E-05
TCERG1	PHB	0.632	0.000697709
TCERG1	NME1	0.691	0.000132612
TCERG1	SRSF1	0.717	5.51E-05
TCERG1	MED13	0.633	0.000683277
TCERG1	NOL11	0.692	0.000128481
TCERG1	WIPI1	-0.627	0.000801769
TCERG1	SRSF2	0.67	0.000248034
TCERG1	EIF4A3	0.621	0.00093137
TCERG1	ALYREF	0.7	9.91E-05
TCERG1	SEH1L	0.644	0.000518037
TCERG1	SNRPD1	0.667	0.000269232
TCERG1	IER3IP1	-0.658	0.000354878
TCERG1	DYM	-0.707	7.85E-05
TCERG1	C18orf32	-0.671	0.000239022
TCERG1	SNRPGP2	0.701	9.61E-05
TCERG1	PIGN	-0.63	0.000732054
TCERG1	SOCS6	-0.665	0.000289079
TCERG1	PTBP1	0.708	7.40E-05
TCERG1	DAZAP1	0.67	0.00024812
TCERG1	CHAF1A	0.664	0.000298579
TCERG1	SAFB	0.682	0.000173042
TCERG1	PRR22	0.646	0.000492327
TCERG1	VMAC	-0.618	0.000990952
TCERG1	LRRC8E	-0.661	0.000324965
TCERG1	ELAVL1	0.693	0.000123539
TCERG1	HNRNPM	0.717	5.53E-05
TCERG1	ILF3	0.672	0.000234149
TCERG1	CTB-55O6.12	0.662	0.000314261
TCERG1	ITGB1P1	-0.623	0.00088156
TCERG1	CHERP	0.63	0.000734156
TCERG1	ZNF101	0.641	0.000550001
TCERG1	ZNF714	0.69	0.000133577
TCERG1	VN1R81P	0.679	0.000188323
TCERG1	ANKRD27	0.67	0.000245521
TCERG1	AC002116.7	0.734	2.96E-05
TCERG1	HNRNPL	0.807	1.09E-06
TCERG1	MAP3K10	-0.649	0.000447654
TCERG1	SNRPA	0.786	3.20E-06
TCERG1	U2AF2	0.707	7.68E-05
TCERG1	ZNF551	0.75	1.59E-05
TCERG1	NOP56	0.71	7.05E-05
TCERG1	MCM8	0.641	0.000561552
TCERG1	GINS1	0.668	0.000266881
TCERG1	RBM12	0.795	2.09E-06
TCERG1	RPRD1B	0.62	0.000955751
TCERG1	SNORA71A	0.761	1.02E-05
TCERG1	SNORA71C	0.719	5.20E-05
TCERG1	DHX35	0.673	0.000224278

TCERG1	TOP1	0.787	3.05E-06
TCERG1	SRSF6	0.664	0.000296861
TCERG1	SERINC3	-0.698	0.000105402
TCERG1	PTTG1IP	-0.667	0.00027282
TCERG1	CECR2	0.677	0.000202779
TCERG1	CDC45	0.687	0.000149922
TCERG1	LL22NC03-2H8.5	0.685	0.000156669
TCERG1	EWSR1	0.619	0.000982251
TCERG1	LGALS1	-0.647	0.000468117
TCERG1	WNT7B	-0.632	0.000697569
TCERG1	TIMP1	-0.649	0.000448925
TCERG1	ZNF711	0.628	0.000770418
TCERG1	LAMP2	-0.628	0.000780775
TCERG1	CETN2	-0.664	0.000294545
TCERG1	HCFC1	0.687	0.000147273
